# Supplementary material for: Offshore wind and wave energy can reduce total installed capacity required in zero-emissions grids
Source: Nat Commun. 2024 Aug 9;15:6826. doi: 10.1038/s41467-024-50040-6 (PMC11316002; doi:10.1038/s41467-024-50040-6)
Supplement: Supplementary file 1 — Supplementary Information [file 41467_2024_50040_MOESM1_ESM.pdf]

Supplementary Information:

Offshore Wind and Wave Energy Can Reduce Total Installed Capacity Required in Zero Emissions Grids

Natalia Gonzalez[a,b,k], Paul Serna-Torre[a,b], Pedro A. Sánchez-Pérez[c], Ryan Davidson[d], Bryan Murray[e], Martin Staadecker[a,f], Julia Szinai[g], Rachel Wei[h], Daniel M. Kammen[i], Deborah A. Sunter[j], Patricia Hidalgo-Gonzalez[a,b,k]

[a]*Mechanical and Aerospace Engineering, University of California San Diego, 9500 Gilman Dr., La Jolla, CA 92093, United States*

[b]*Center for Energy Research, University of California San Diego, 9500 Gilman Dr., La Jolla, CA 92093, United States*

[c]*School of Engineering, University of California Merced, 5200 Lake Rd, Merced, CA, 95340, United States*

[d]*CalWave, 1595 Portland Ave, Berkeley, CA 94707, United States*

[e]*University of Oviedo, C. San Francisco, Oviedo 3, 33003, Asturias, Spain*

[f]*Division of Engineering Science, Un, ON M5S 1A1, Ontario, Canada*

[g]*Lawrence Berkeley National Laboratory, 1 Cyclotron Rd, Berkeley, 94720, CA, United States*

[h]*Computer Science and Engineering, University of California San Diego, 9500 Gilman Dr., La Jolla, CA 92093, United States*

[i]*Renewable and Appropriate Energy Laboratory, Energy Resources Group, University of California Berkeley, 339 Giannini Hall, Berkeley, CA 94720, United States*

[j]*Civil and Environmental Engineering, Tufts University, 200 College Ave, Medford, MA, 02155, United States*

[k]*Corresponding authors: Patricia Hidalgo-Gonzalez, phidalgogonzalez@ucsd.edu, and Natalia Gonzalez, n7gonzalez@ucsd.edu*

Contents

Contents **1**

List of Figures **3**

List of Tables **3**

1 Supplementary Methodological Information 5
- Candidate Projects Design 5
- Wave Energy Capacity Factors 8

2 Supplementary Visualization: Scenario Cost Targets 12

3 Supplementary Graphical Results 13
- All Investment Periods 13
- Investment Period 2050 Only 19

4 Supplementary Numerical Results 20
- Installed Capacity 20
- Dispatch and Curtailment 23
- Transmission 23
- Collocation 24
- System Costs 26

5 Supplementary Model Information: SWITCH Data 26
- Geographic and Temporal Resolution 27
- Generators 28
  - Existing Generation Data 28
  - Candidate Generators 29
  - Technology Assumptions On Lifetime, Capacity Factors, Efficiencies 33
- Transmission 35
  - Existing and Candidate Transmission 35
- Costs 37
  - Overnight Capital Cost, Variable O&M Cost, Fixed O&M Cost 37
  - Connection Costs 40
  - Fuel Costs and Emissions 40
  - Transmission Costs 41
- Load 41

Policies 42
Renewable Portfolio Standards 42
Carbon Cap 42
6 Supplementary SWITCH Mathematical Formulation 43
Objective Function 43
Operational Constraints 44
Power balance 44
Dispatch 44
Investment Constraints 45
SWITCH Modules 45
Treatment of time 45
Financial components 46
Load zones and power injection/withdrawal 46
Energy sources 47
Dispatch components 48
Fuel costs 49
Hydropower components 52
Storage components 52
Carbon cap components 56
Minimum technology requirements 57
Enforcing a solar to wind capacity ratio 57

Bibliography **58**

List of Figures

1 Ocean depth contour lines along U.S. West Coast 6
2 U.S. marine zones 7
3 Interpolated capacity factor visualization 12
4 Wave energy overnight and O&M cost targets assumed by scenario 12
5 Offshore wind energy overnight and O&M cost targets assumed by scenario . . . 13
6 Generation capacity mix in each investment period for four edge-case scenarios . 14
7 Total annual renewable curtailment (TWh) in each investment period for four edge-case scenarios 15
8 Percentage of total annual energy that is curtailed in each investment period for four edge-case scenarios 16
9 Total built transmission (GW) in each investment period for four edge-case scenarios 17
10 Total installed transmission capacity (GW) in each investment period for four edge-case scenarios 18
11 2050 daily dispatch profiles for the entire Western Interconnection for four edge-case scenarios 19
12 SWITCH-WECC load zone boundaries 28
13 SWITCH-WECC transmission topology 36

List of Tables

1 Sites of industry interest removed from consideration for candidate project areas 5
2 Capacity factor sampling error analysis 8
3 2050 offshore wind energy installed capacity in each scenario 20
4 2050 offshore wind energy percent installed capacity in each scenario 20
5 2050 wave energy installed capacity in each scenario 21
6 2050 wave energy percent installed capacity in each scenario 21
7 2050 land-based wind energy installed capacity in each scenario 21

8 2050 total generation installed capacity in the Western Interconnection in each scenario . . . 22
9 2050 solar energy installed capacity in each scenario . . . 22
10 2050 energy storage installed capacity in each scenario . . . 22
11 2050 ratio of generation to load for California in each scenario . . . 23
12 2050 energy curtailment in the Western Interconnection in each scenario . . . 23
13 2050 transmission installed capacity in each scenario . . . 23
14 Number of sites chosen for collocation in 2050 in each scenario . . . 24
15 Percent of installed offshore wind and/or wave energy farms that have collocation of both technologies in 2050 in each scenario . . . 24
16 Most frequently collocated sites . . . 25
17 WECC load zone abbreviation descriptions for load zones with the most frequently collocated sites . . . 25
18 Total system itemized costs in each scenario . . . 26
19 WECC total available capacity of existing and candidate generation . . . 31
20 Technology assumptions for existing and candidate generators . . . 34
21 Average capital, fixed O&M, and variable O&M costs by investment period for candidate generators . . . 37
22 Average fuel costs across load zones, by investment period . . . 41
23 WECC-wide and California carbon cap average by investment period . . . 43
24 Emissions intensity of fuel-based energy sources . . . 43
25 Model components defined in the `timescales` module . . . 46
26 Model components defined in the `financials` module . . . 46
27 Model components defined in the `balancing.load_zones` module . . . 47
28 Model components defined in the `energy_sources.properties` module . . . 47
29 Model components defined in the `generators.core.build` module . . . 48
30 Model components defined in the `generators.core.dispatch` module . . . 49
31 Model components defined in the `generators.core.no_commit` module . . . 49
32 Model components defined in the `energy_sources.fuel_costs.simple` module 49
33 Model components defined in the `energy_sources.fuel_costs.markets` module 50
34 Model components defined in the `transmission.transport.build` module . . . 51
35 Model components defined in the `transmission.transport.dispatch` module . 52
36 Model components defined in the `generators.extensions.hydro_simple` module 52
37 Model components defined in the `generators.extensions.storage` module . . 53
38 Model components defined in the `policies.carbon_policies` module . . . 56
39 Model components defined in the `policies.min_per_tech` module . . . 57
40 Model components defined in the `policies.wind_to_solar_ratio` module . . . 57

1 Supplementary Methodological Information

This section of the Supplementary Information provides additional details about the methodology, including information about the design of candidate projects and the generation of wave energy capacity factors.

Candidate Projects Design

Table 1: Sites of industry interest removed from consideration for candidate project areas. Source data are provided as a Source Data file.

Site Name	Latitude	Longitude
Port Hueneme 2	33.283	119.613
Newport	39.576	-123.815
Dogtown 1	37.818	-123.098
Lompoc 7	34.138	-120.551

The shape files for the Call Areas are downloaded from the Bureau of Ocean Energy Management (BOEM) [1]. The shape files for the Marine Protected Areas (MPAs) are downloaded from the Bureau of Ocean Energy Management (BOEM) [1]. The shape files for the MPA zones [2] and military restricted areas [3] are overlaid with the sites of industry interest to determine which sites are not viable. All figures depicting US States display the shape file from [4].

Marine restrictions that are not considered in the site filtering process include shipping routes, touristic sites, non-protected aquaculture zones, areas of particular landscape value, undersea archaeological sites (other than United Nations Educational, Scientific and Cultural Organization (UNESCO) World Heritage Marine Sites (WHMSs)), or existing underwater cables and pipelines. Shipping routes, touristic sites, non-protected aquaculture zones, and areas of particular landscape value are excluded from consideration because this study is focused on the year 2050, by which many of these classifications may change or adjust to accommodate new marine activities. Undersea archaeological sites other than UNESCO WHMSs are excluded due to limited data availability. Existing underwater cables and pipelines are also excluded since offshore wind planners acknowledge that "it is almost inevitable that export cables will need to cross other subsea assets between the offshore wind farm and the shore landing" and that developers "must design a suitable crossing methodology and obtain a crossing agreement from the owner of the asset that will be crossed" [5].

Figure 1 shows the water depth contours on the U.S. West Coast in meters. We do not enforce a maximum water depth on the offshore wind and wave energy candidate projects because it is uncertain what water depths will be possible to install marine energy devices in the year 2050 due to technological advancements over the coming decades. The study in [6]

shows deep water floating offshore wind turbines in 600m to 900m depths. Only 8 of the 101 candidate project areas have any portions of their areas beyond the 900m depth contour. Furthermore, the official Offshore Wind Call Areas that represent offshore wind energy sites of interest as identified by BOEM are between 200m and 1300m deep. Only 5 of the 101 candidate project areas have any portions of their areas beyond the 1300m depth contour .The water depth data from [7], as shown in in figure 1 only shows contours up to 1500m depths. Only 5 of the 101 candidate project areas have any portions of their areas beyond the 1500m depth contour.

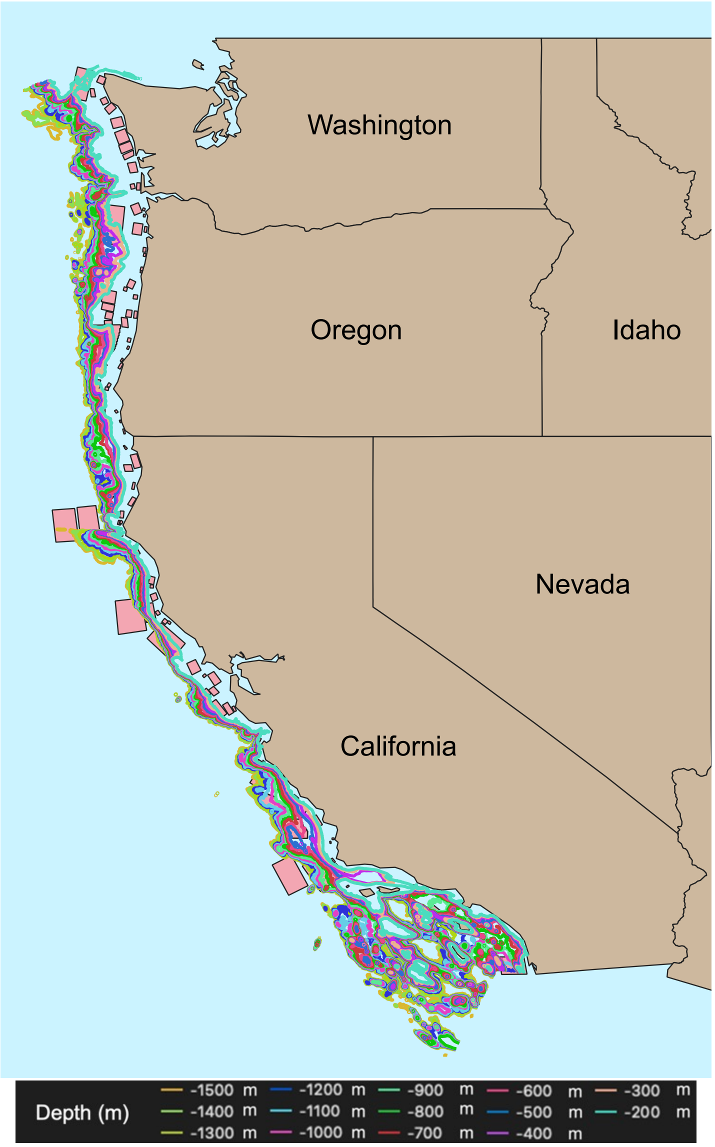

Figure 1: Ocean depth contour lines along U.S. West Coast. The legend shows ocean depth in meters.

Figure 2 shows the EEZ, the Contiguous Zone, and the Territorial Sea with respect to the candidate projects [8]. Only two candidate projects fall completely outside of the Territorial Sea, which is the maritime zone which the U.S. exercises sovereignty. All candidate project areas fall within the Exclusive Economic Zone (EEZ) of the U.S. The EEZ extends 200 nautical miles from the Territorial Sea baseline of the U.S., and it is the zone which the U.S. has "sovereign rights for the purpose of exploring, exploiting, conserving and managing natural resources, whether living and nonliving, of the seabed and subsoil and the superjacent waters and with regard to other activities for the economic exploitation and exploration of the zone, such as the production of energy from the water, currents and winds" [8].

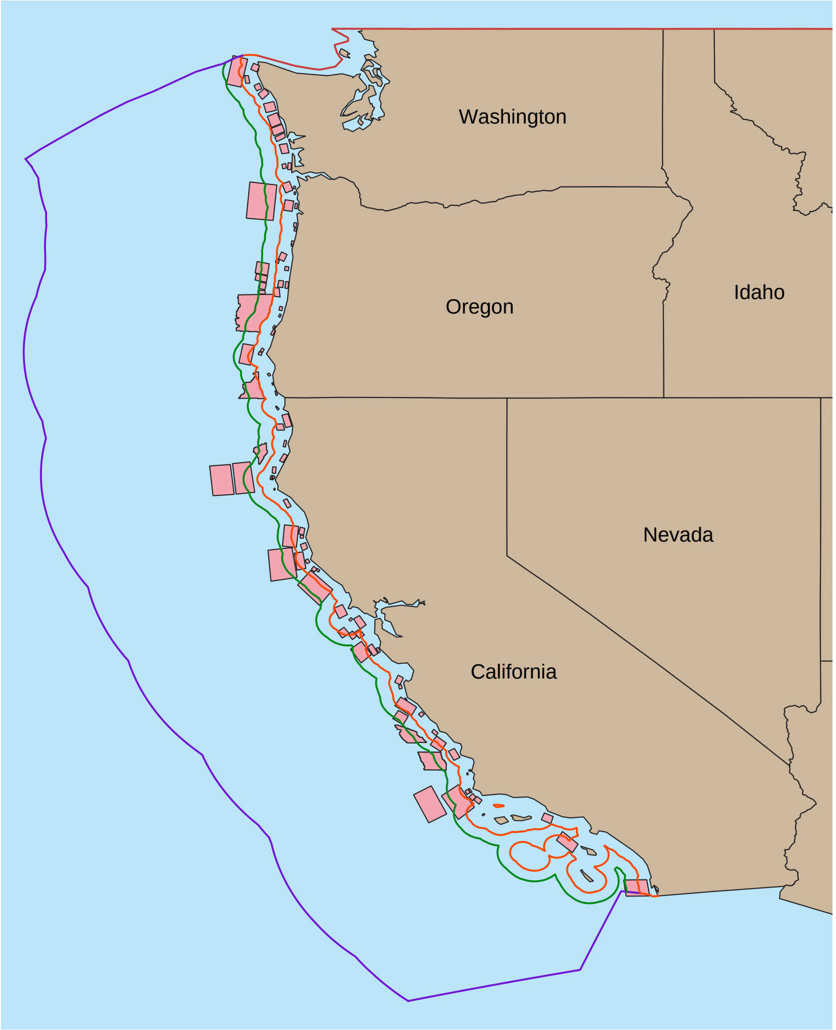

Figure 2: Exclusive Economic Zone (EEZ) (blue line), Contiguous Zone (green line), and the Territorial Sea (red line) boundaries on the U.S. West Coast with respect to the candidate project areas (pink polygons) for offshore wind and wave energy.

Wave Energy Capacity Factors

We identify 89,650 coordinates from the U.S. Department of Energy (DOE) Water Power Technology Office's (WPTO) U.S. Wave dataset [9], [10] that overlap with the candidate project areas. We overlay these coordinates with the candidate project areas in QGIS to identify 89,650 overlapping coordinates. Then, we use 3-hour time resolution time series of wave characteristics for the year 2006 corresponding to every ten (to reduce download time) of the 89,650 coordinates from [9]. A total of 8,811 coordinates are successfully downloaded. To demonstrate that it is not necessary to use all 89,650 overlapping coordinates in the calculation of the average capacity factor time series for each site, we compare the capacity factor time series associated to the coordinates in each area to the resulting average time series. The mean squared error (MSE) between the raw capacity factor time series and the averaged capacity factor time series is calculated for the individual coordinates within each site. The average of these MSE for each site is shown in Table 2, along with the average of the absolute difference between the standard deviation in each raw capacity factor time series and the standard deviation of the averaged capacity factor time series for each site. The average of the MSE and standard deviation metrics across all sites are low (6.03E-04 and 4.73E-04, respectively), which suggests that the wave data capacity factors do not vary significantly across coordinates that are near each other. Therefore, it is reasonable to assume that downloading the capacity factor time series of all 89,650 overlapping coordinates would not change the average capacity times series calculated for each site significantly.

Table 2: Error analysis to justify taking every 10 points (rather than all points) and taking an average time series of wave energy capacity factors per candidate project area or 'site'. Column 3 shows the average mean squared error (MSE) between the unaveraged. Source data are provided as a Source Data file.

Site Name	Site ID	Average MSE	Average Absolute Error in Standard Deviation
Neah Bay 1	0	1.51E-03	1.14E-03
Neah Bay 2	1	7.24E-04	6.11E-04
La Push 1	2	1.30E-04	1.14E-04
La Push 2	3	4.44E-04	2.59E-04
La Push 3	4	8.69E-04	4.69E-04
Queets	5	9.80E-04	5.30E-04
Taholah	6	1.36E-03	7.48E-04
Ocean Shores 1	7	1.12E-03	6.12E-04
Ocean Shores 2	8	1.19E-03	7.76E-04
Grayland	9	1.55E-03	8.98E-04
Long Beach 1	10	2.29E-04	1.59E-04
Long Beach 2	11	2.92E-04	2.91E-04
Seaside 1	12	1.51E-04	1.32E-04
Seaside 2	13	1.85E-04	1.16E-04

Table 2: (continued)

Site Name	Site ID	Average MSE	Average Absolute Error in Standard Deviation
Cannon Beach	14	2.27E-04	1.35E-04
Manzanita	15	5.00E-04	5.96E-04
Rockaway Beach 1	16	1.99E-04	1.59E-04
Rockaway Beach 2	17	2.64E-04	1.70E-04
Tierra del Mar	18	2.88E-04	2.01E-04
Pacific City	19	2.46E-04	2.04E-04
Lincoln City	20	3.25E-04	2.58E-04
Lincoln Beach	21	3.72E-04	2.69E-04
Newport 1	22	1.26E-04	1.11E-04
Newport 2	23	8.28E-05	9.88E-05
Waldport 1	24	1.18E-04	8.65E-05
Waldport 2	25	1.71E-04	1.93E-04
Searose Beach 1	26	1.63E-04	1.55E-04
Searose Beach 2	27	7.54E-05	6.80E-05
Heceta Beach 1	28	3.30E-04	2.08E-04
Heceta Beach 2	29	1.28E-04	1.14E-04
Heceta Beach 3	30	6.47E-05	7.04E-05
Heceta Beach 4	31	7.42E-05	7.18E-05
Reedsport	32	7.34E-05	5.08E-05
Lakeside	33	6.50E-05	5.66E-05
Coos Bay	34	2.99E-04	2.83E-04
Bandon	35	2.83E-03	7.23E-04
Denmark	36	8.60E-05	7.30E-05
Sixes 1	37	1.04E-04	6.06E-05
Sixes 2	38	4.68E-04	5.43E-04
Wedderburn	39	9.11E-04	6.30E-04
Pistol River	40	1.60E-04	1.87E-04
Brookings	41	4.04E-04	2.65E-04
Crescent City 1	42	3.19E-04	2.31E-04
Crescent City 2	43	1.84E-03	1.56E-03
Klamath 1	44	4.13E-03	1.51E-03
Klamath 2	45	3.51E-04	2.46E-04
Big Lagoon	46	3.19E-04	1.84E-04
Eureka	47	1.18E-03	8.21E-04
Port Kenyon	48	8.28E-04	7.76E-04
Capetown 1	49	1.09E-03	1.34E-03
Capetown 2	50	1.14E-03	1.33E-03
Capetown 3	51	1.85E-04	2.95E-04

Table 2: (continued)

Site Name	Site ID	Average MSE	Average Absolute Error in Standard Deviation
Capetown 4	52	4.57E-04	6.66E-04
Capetown 5	53	3.45E-04	5.67E-04
Petrolia	54	5.14E-05	5.48E-05
Shelter Cove	55	1.43E-03	1.11E-03
Coos Bay Call Area	56	1.28E-03	1.23E-03
Fort Bragg	57	4.74E-04	2.86E-04
Caspar 1	58	1.51E-04	8.72E-05
Caspar 2	59	8.14E-04	7.37E-04
Whitesboro	60	4.88E-04	2.53E-04
Flumeville 1	61	1.09E-03	1.21E-03
Flumeville 2	62	4.73E-04	4.60E-04
Flumeville 3	63	2.88E-04	1.35E-04
Anchor Bay	64	7.67E-04	4.43E-04
Gualala	65	6.29E-04	3.92E-04
Fort Ross	66	1.50E-03	1.21E-03
Point Reyes Station	67	7.66E-04	4.05E-04
Brookings_Call	68	1.46E-03	1.62E-03
Dogtown 2	69	1.08E-04	8.05E-05
Stinson Beach	70	1.87E-03	1.39E-03
Moss Beach 1	71	2.03E-04	2.44E-04
Moss Beach 2	72	4.58E-04	2.70E-04
Moss Beach 3	73	5.60E-04	2.97E-04
Pescadero 1	74	2.06E-04	1.46E-04
Pescadero 2	75	8.23E-04	4.88E-04
Pescadero 3	76	9.16E-04	4.07E-04
Pacific Grove	77	5.44E-04	2.59E-04
Carmel Highlands	78	5.98E-05	3.82E-05
Big Sur 1	79	1.66E-04	1.58E-04
Big Sur 2	80	1.18E-03	8.23E-04
Lucia 1	81	7.24E-04	3.46E-04
Lucia 2	82	3.20E-04	3.02E-04
San Simeon 1	83	5.36E-04	2.22E-04
San Simeon 2	84	4.55E-04	3.61E-04
Baywood-Los Osos	85	5.56E-04	2.98E-04
Guadalupe 1	86	3.31E-04	2.04E-04
Guadalupe 2	87	6.28E-05	2.01E-05
Lompoc 1	88	1.09E-04	4.98E-05
Lompoc 2	89	1.72E-04	8.97E-05

Table 2: (continued)

Site Name	Site ID	Average MSE	Average Absolute Error in Standard Deviation
Lompoc 3	90	3.84E-04	1.93E-04
Lompoc 4	91	9.30E-04	4.01E-04
Lompoc 5	92	9.89E-04	9.66E-04
Lompoc 6	93	4.40E-04	6.98E-04
Humboldt Call Area	94	6.52E-04	6.30E-04
Lompoc 8	95	1.90E-04	3.23E-04
Port Hueneme 1	96	6.47E-04	2.98E-03
Torrance	97	6.15E-04	1.12E-03
Morro Bay Call Area	98	5.83E-04	5.25E-04
San Diego	99	4.42E-04	5.89E-04
Diablo Canyon Call Area	100	9.46E-04	7.62E-04
**AVERAGE across all sites**		**6.03E-04**	**4.73E-04**

We use the U.S. Department of Energy (DOE) Water Power Technology Office's (WPTO) U.S. Wave dataset [9], [10] to compute capacity factor time series for each candidate project area. This dataset has a 3-hour time resolution, but SWITCH requires hourly capacity factors for variable renewable energy sources as an input parameter. We linearly interpolate to convert the time resolution of the dataset from 3-hour to 1-hour resolution. To verify that the linear interpolation does not introduce substantial error, we compare 1-hour data from the virtual buoys of [9] with the linearly interpolated (3-hour to 1-hour) data use in this study. The virtual buoy hourly dataset only contains 92 coordinates with wave characteristic time series along the U.S. West Coast. Each of the 92 coordinates is matched with the closest (geographically) coordinate from the 3-hour dataset. Two sets of coordinates are discarded due to several 1-hour coordinates matching with the same 3-hour coordinate. Out of the remaining 90 coordinate sets, 20 are discarded due to an error in the 1-hour dataset which causes significant wave height and energy period profiles to have constant zero values through the entirety of 2006. Significant wave height and energy period profiles for the year 2006 are downloaded from the 1-hour and 3-hour datasets for the remaining 70 coordinate pairs. The capacity factor is calculated for each significant wave height and energy period pair at each timepoint for each dataset. The 3-hour dataset is interpolated to a 1-hour time resolution, and the capacity factors of the interpolated dataset are compared to the capacity factors of the 1-hour dataset. The average MSE between the capacity factors of the 3-hour interpolated to 1-hour dataset and the 1-hour dataset for the year 2006 across all 70 locations is 0.0044. Figure 3 shows the hourly capacity factors of the two datasets for a random pair of matched coordinates from the list of coordinates used in this study.

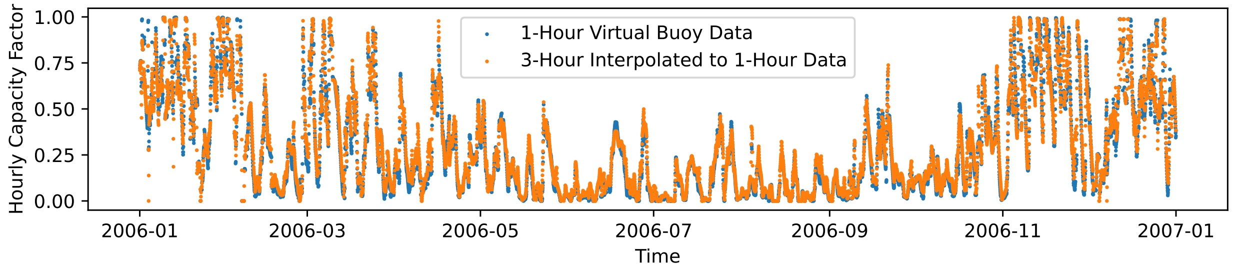

Figure 3: Capacity factors from the 1-hour virtual buoy dataset vs. capacity factors from the 3-hour interpolated to 1-hour dataset used in this study. Source data are provided as a Source Data file.

2 Supplementary Visualization: Scenario Cost Targets

This section of the Supplementary Information contains visualizations of the cost targets used in each scenario of this study.

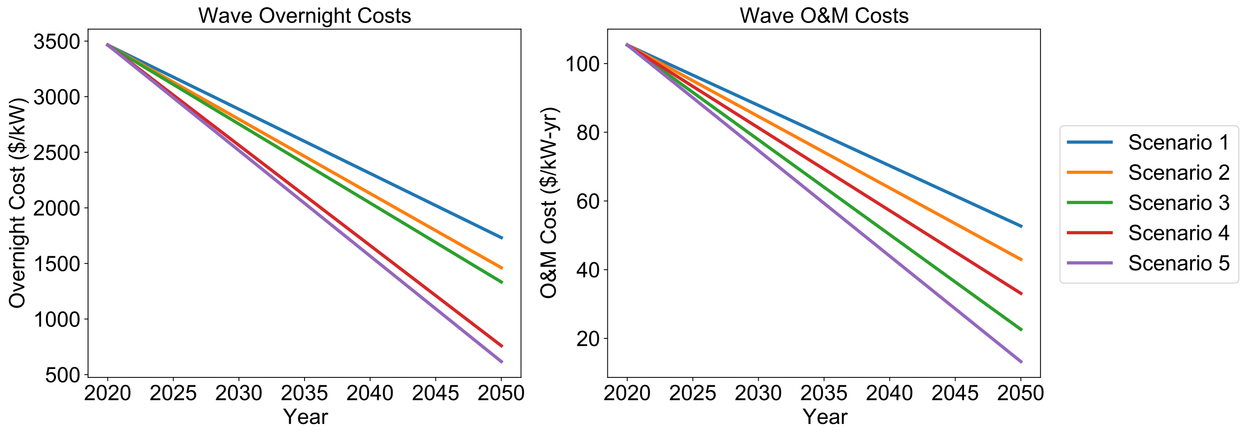

Figure 4: Wave energy overnight and O&M cost targets assumed by scenario. Note that although cost targets are only shown for scenario set 1-5, scenario sets 6-10, 11-15, 16-20, and 21-25 assume the same wave energy cost targets. Source data are provided as a Source Data file.

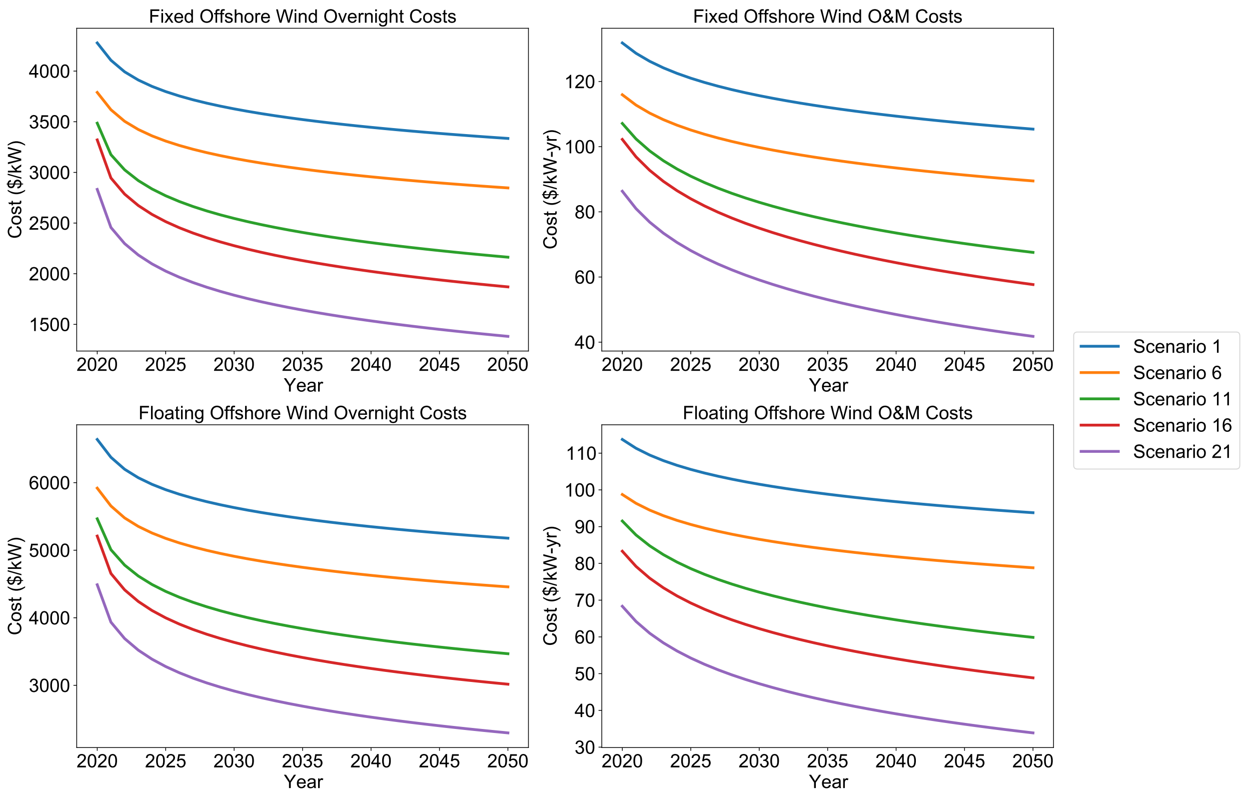

Figure 5: Offshore wind energy overnight and O&M cost targets assumed by scenario. Note that although cost targets are only shown for scenario set (1,6,11,16,21), scenario sets (2,7,12,17,22), (3,8,13,18,23), (4,9,14,19,24), and (5,10,15,20,25) assume the same wave energy cost targets. Source data are provided as a Source Data file.

3 Supplementary Graphical Results

This section of the Supplementary Information contains graphical results related to the scenarios in this study.

All Investment Periods

The following subsection presents graphical results for all investment periods (2020, 2030, 2040, and 2050) for each of the four edge-case scenarios, which represent the most extreme cost assumptions for each of the marine technologies. As a reminder, scenario 1 has the most conservative offshore wind and wave energy costs, scenario 5 has the most conservative offshore wind energy costs and most optimistic wave energy costs, scenario 21 has the most optimistic offshore wind energy costs and most conservative wave energy costs, and scenario 25 has the most optimistic offshore wind and wave energy costs.

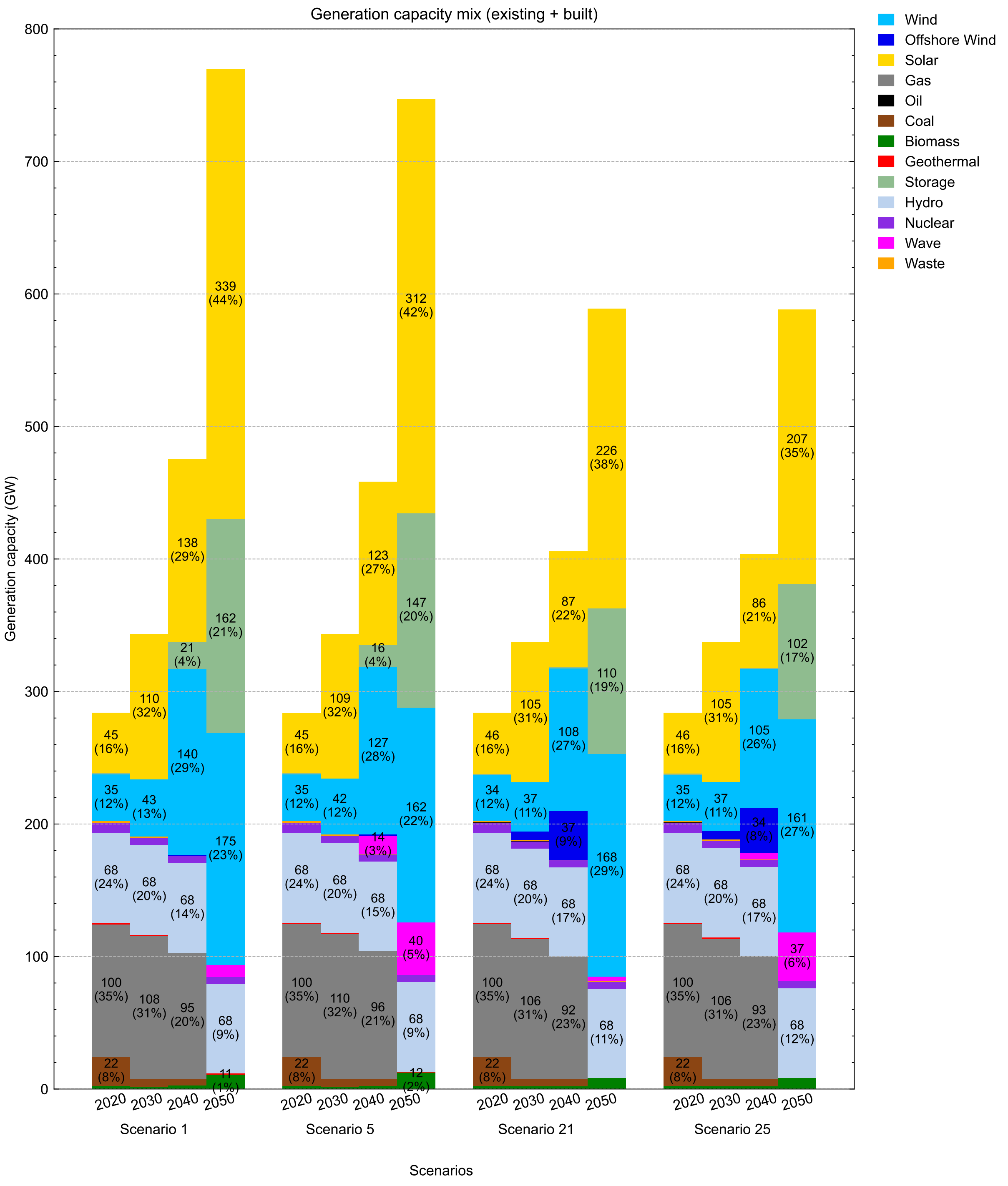

Figure 6: Generation capacity mix in each investment period for four edge-case scenarios. Source data are provided as a Source Data file.

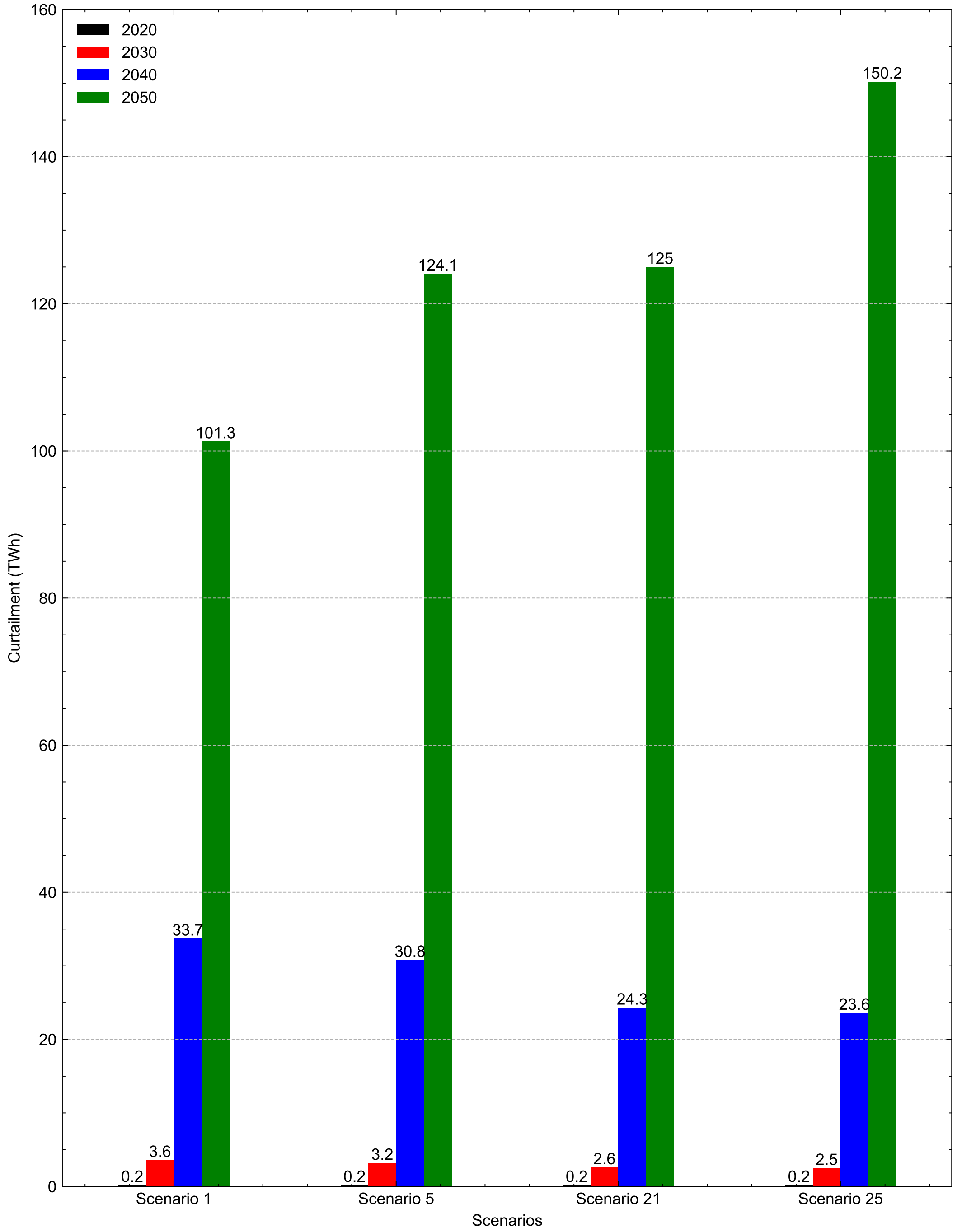

Figure 7: Total annual renewable curtailment (TWh) in each investment period for four edge-case scenarios. Source data are provided as a Source Data file.

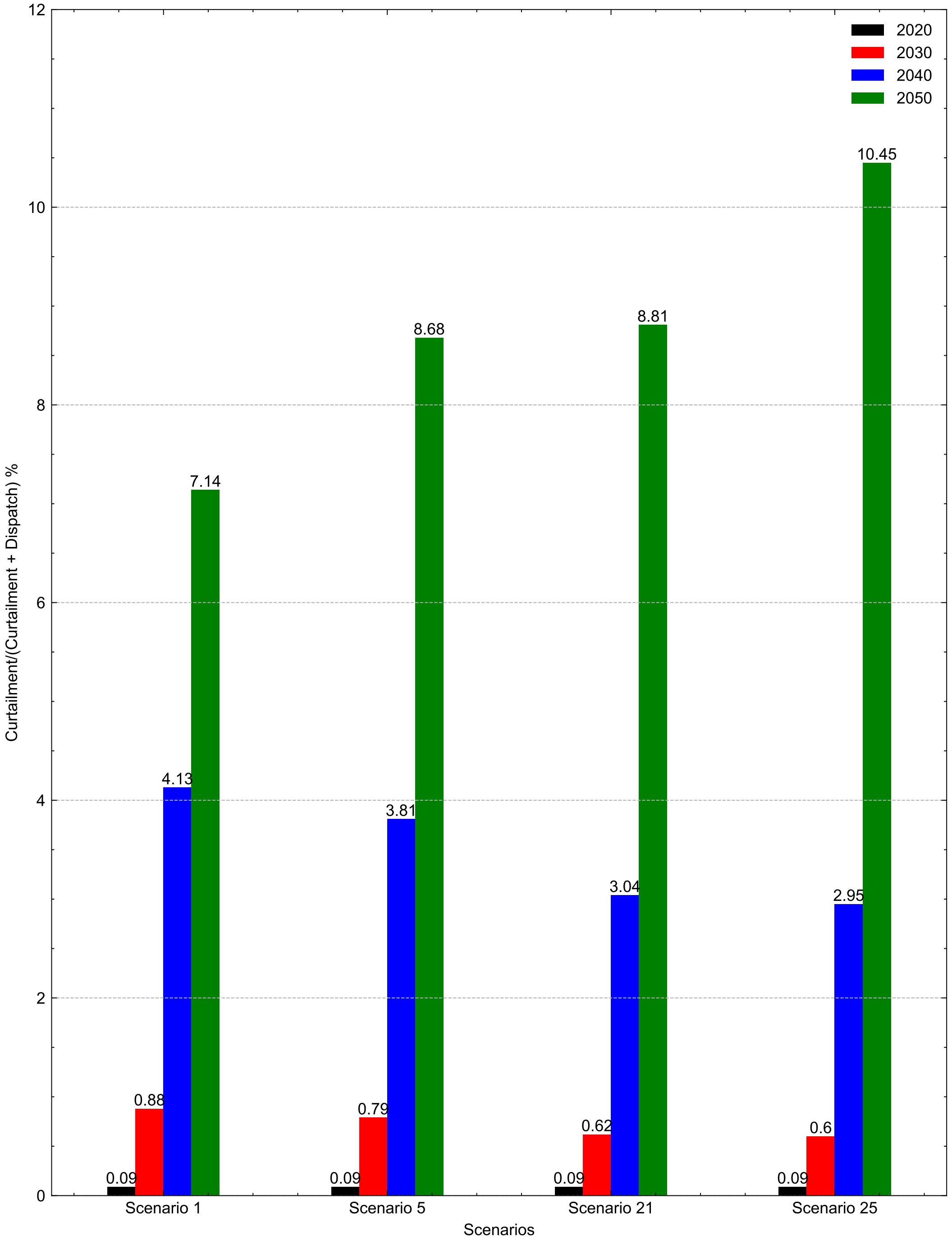

Figure 8: Percentage of total annual energy (renewable curtailment + total dispatch) that is curtailed in each investment period for four edge-case scenarios. Source data are provided as a Source Data file.

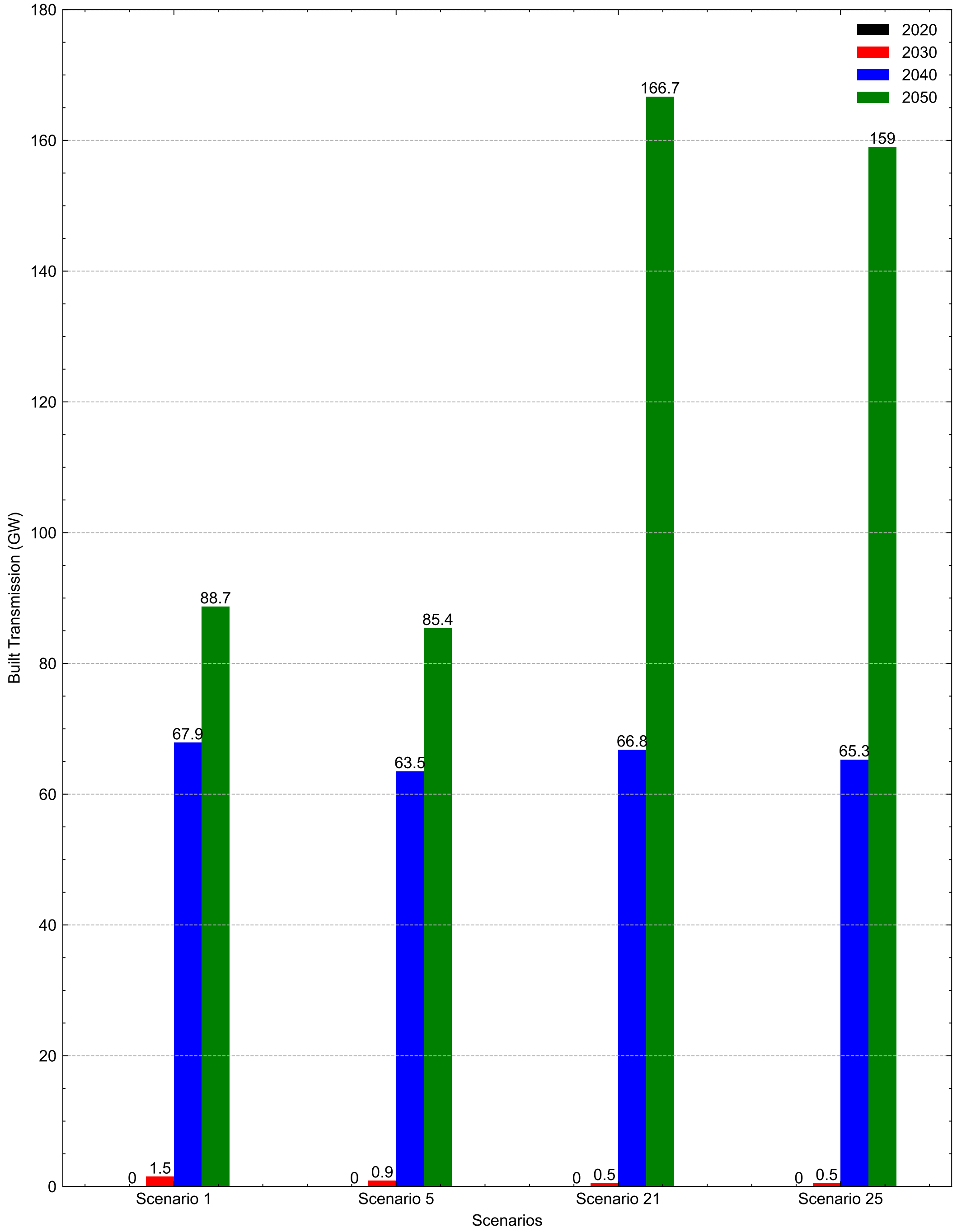

Figure 9: Total built transmission (GW) in each investment period for four edge-case scenarios. Source data are provided as a Source Data file.

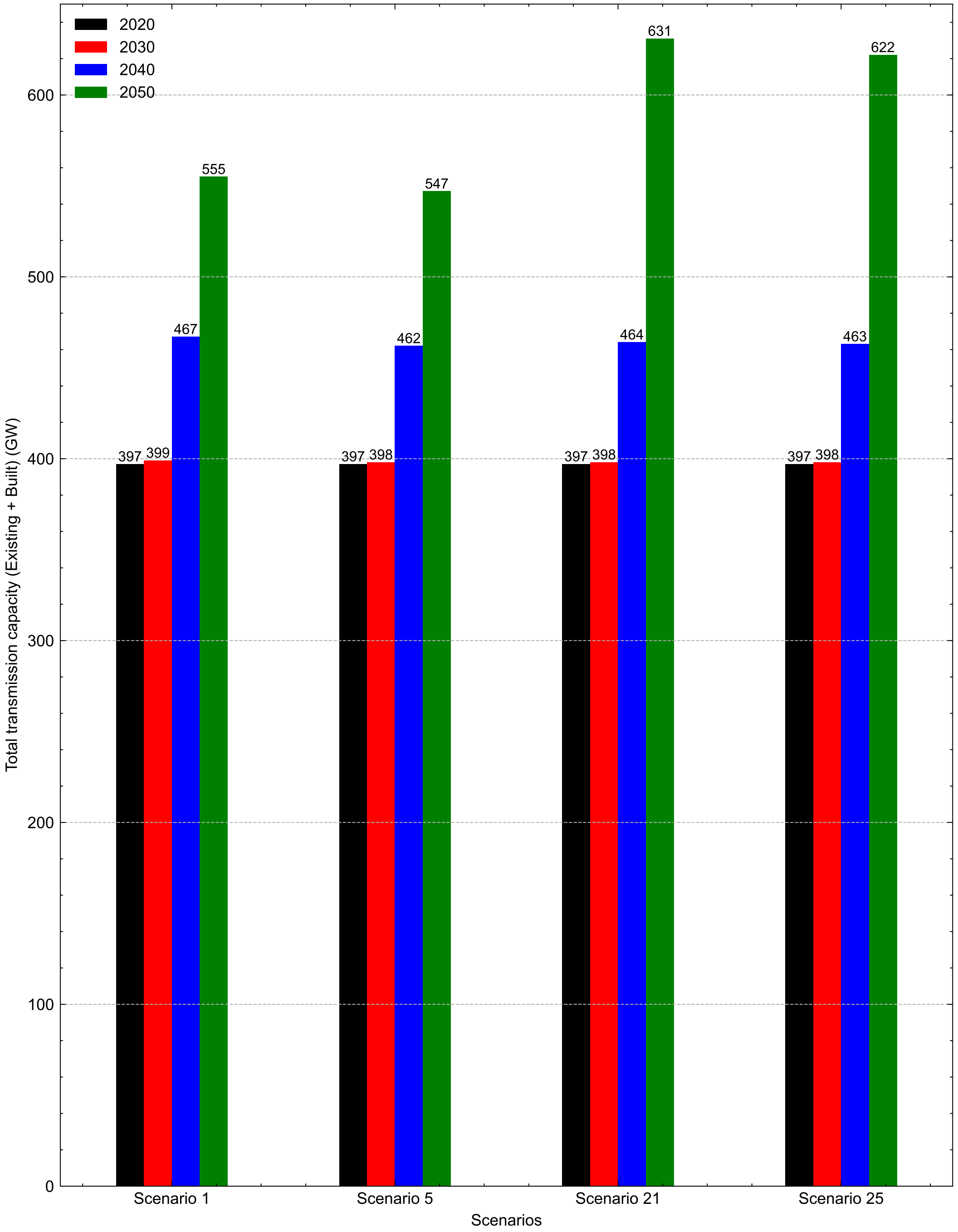

Figure 10: Total installed transmission capacity (GW) in each investment period for four edge-case scenarios. Source data are provided as a Source Data file.

Investment Period 2050 Only

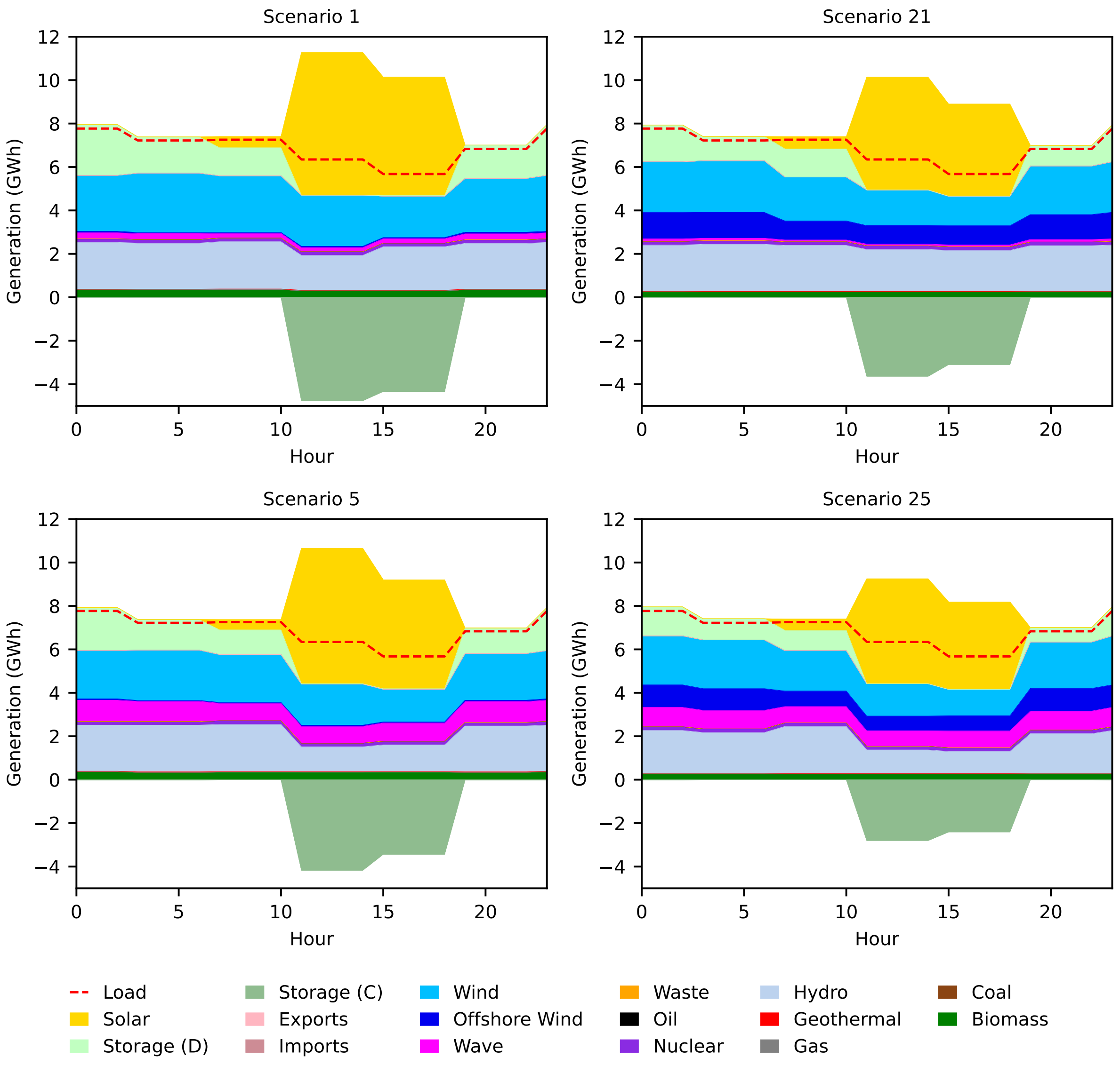

Figure 11: 2050 daily (peak day Dec.) dispatch profiles for the entire Western Interconnection for four edge-case scenarios. Similar to the coastal load zones, the daily dispatch profiles of the entire Western Interconnection show relatively constant offshore wind (blue) and wave power (magenta) generation, decreased dispatch of solar energy (yellow) and energy storage (light green) with increased dispatch of offshore wind (blue) and wave energy (magenta). Source data are provided as a Source Data file.

4 Supplementary Numerical Results

The following section presents tables and figures containing detailed numerical results described in section 3 of the main body. Note that in tables 5-15, only floating offshore wind 2050 overnight costs are used to label how the offshore wind cost targets vary along the x-axis of the table, although every scenario has a mix of fixed and floating offshore wind projects that have different cost assumptions. The reason for this is simply for visual simplification. Both fixed and floating offshore wind cost targets vary in the same way across the scenarios. This convention is used so the reader may understand which cost target corresponds to which scenario, and how "advanced" or "conservative" that cost target is relative to the others.

Installed Capacity

Table 3: 2050 offshore wind energy installed capacity in each scenario (GW). Source data are provided as a Source Data file.

		Floating Offshore Wind 2050 Overnight Costs ($/kW)				
		$5,178	$4,458	$3,468	$3,017	$2,296
Wave Energy 2050 Overnight Costs ($/kW)	$1,733	2.41	4.00	23.01	35.74	58.91
	$1,462	2.36	3.80	20.83	33.61	57.96
	$1,333	2.36	3.80	18.15	29.64	56.05
	$760	2.09	3.55	17.42	26.27	54.54
	$618	2.05	3.02	15.90	25.69	51.84

Table 4: 2050 offshore wind energy installed capacity in each scenario (as a percent of total generation capacity in the Western Interconnection). Source data are provided as a Source Data file.

		Floating Offshore Wind 2050 Overnight Costs ($/kW)				
		$5,178	$4,458	$3,468	$3,017	$2,296
Wave Energy 2050 Overnight Costs ($/kW)	$1,733	0.31	0.52	3.21	5.22	9.09
	$1,462	0.31	0.50	2.91	4.92	9.01
	$1,333	0.31	0.51	2.53	4.31	8.73
	$760	0.28	0.48	2.45	3.82	8.55
	$618	0.27	0.41	2.22	3.73	8.10

Table 5: 2050 wave energy installed capacity in each scenario (GW). Source data are provided as a Source Data file.

		Floating Offshore Wind 2050 Overnight Costs ($/kW)				
		$5,178	$4,458	$3,468	$3,017	$2,296
Wave Energy 2050 Overnight Costs ($/kW)	$1,733	8.86	8.86	7.64	5.59	3.67
	$1,462	19.33	18.44	13.97	11.33	7.64
	$1,333	24.96	24.93	24.34	21.14	13.52
	$760	35.51	35.51	34.50	33.99	29.60
	$618	39.55	39.55	39.55	38.88	36.58

Table 6: 2050 wave energy installed capacity in each scenario (as a percent of total generation capacity in the Western Interconnection). Source data are provided as a Source Data file.

		Floating Offshore Wind 2050 Overnight Costs ($/kW)				
		$5,178	$4,458	$3,468	$3,017	$2,296
Wave Energy 2050 Overnight Costs ($/kW)	$1,733	1.15	1.15	1.07	0.82	0.57
	$1,462	2.54	2.43	1.95	1.66	1.19
	$1,333	3.30	3.31	3.40	3.08	2.11
	$760	4.74	4.76	4.84	4.94	4.64
	$618	5.29	5.30	5.53	5.64	5.72

Table 7: 2050 land-based wind energy installed capacity in each scenario (GW). Source data are provided as a Source Data file.

		Floating Offshore Wind 2050 Overnight Costs ($/kW)				
		$5,178	$4,458	$3,468	$3,017	$2,296
Wave Energy 2050 Overnight Costs ($/kW)	$1,733	174.92	174.09	175.80	177.30	168.06
	$1,462	169.25	169.10	172.67	173.01	168.97
	$1,333	165.89	165.38	161.41	162.98	167.97
	$760	164.34	163.43	159.74	158.65	162.69
	$618	162.10	161.65	157.22	156.82	161.09

Table 8: 2050 total generation installed capacity in the Western Interconnection in each scenario (GW). Source data are provided as a Source Data file.

		Floating Offshore Wind 2050 Overnight Costs ($/kW)				
		$5,178	$4,458	$3,468	$3,017	$2,296
Wave Energy 2050 Overnight Costs ($/kW)	$1,733	771.20	767.38	716.72	684.39	647.87
	$1,462	760.77	758.01	716.17	683.36	643.64
	$1,333	755.63	752.21	716.98	687.17	641.97
	$760	749.21	745.85	712.29	688.33	637.92
	$618	748.09	745.82	715.17	688.97	639.96

Table 9: 2050 solar energy installed capacity in each scenario (GW). Source data are provided as a Source Data file.

		Floating Offshore Wind 2050 Overnight Costs ($/kW)				
		$5,178	$4,458	$3,468	$3,017	$2,296
Wave Energy 2050 Overnight Costs ($/kW)	$1,733	339.41	336.21	288.45	260.73	226.20
	$1,462	329.39	327.03	286.83	260.10	220.30
	$1,333	324.53	321.46	290.97	265.41	217.28
	$760	313.93	311.18	282.60	262.05	207.48
	$618	312.27	310.18	284.07	260.42	207.11

Table 10: 2050 energy storage installed capacity in each scenario (GW). Source data are provided as a Source Data file.

		Floating Offshore Wind 2050 Overnight Costs ($/kW)				
		$5,178	$4,458	$3,468	$3,017	$2,296
Wave Energy 2050 Overnight Costs ($/kW)	$1,733	161.57	160.39	138.97	122.78	109.74
	$1,462	156.10	155.43	138.84	122.98	107.52
	$1,333	153.35	152.23	139.17	125.55	105.93
	$760	148.00	147.03	134.51	124.85	102.44
	$618	146.64	146.12	134.43	124.35	101.81

Dispatch and Curtailment

Table 11: 2050 ratio of generation to load for California in each scenario. Source data are provided as a Source Data file.

		Floating Offshore Wind 2050 Overnight Costs ($/kW)				
		$5,178	$4,458	$3,468	$3,017	$2,296
Wave Energy 2050 Overnight Costs ($/kW)	$1,733	0.87	0.87	0.89	0.91	0.93
	$1,462	0.89	0.89	0.90	0.93	0.94
	$1,333	0.90	0.89	0.92	0.92	0.94
	$760	0.91	0.91	0.93	0.94	0.94
	$618	0.91	0.91	0.92	0.94	0.94

Table 12: 2050 energy curtailment in the Western Interconnection in each scenario (TWh). Source data are provided as a Source Data file.

		Floating Offshore Wind 2050 Overnight Costs ($/kW)				
		$5,178	$4,458	$3,468	$3,017	$2,296
Wave Energy 2050 Overnight Costs ($/kW)	$1,733	101.31	100.65	106.12	112.74	125.02
	$1,462	109.32	104.97	108.07	113.46	127.47
	$1,333	113.22	111.02	112.91	109.51	132.33
	$760	122.55	120.92	122.12	118.74	145.95
	$618	124.15	124.45	123.70	120.15	150.22

Transmission

Table 13: 2050 transmission installed capacity in each scenario (GW). Source data are provided as a Source Data file.

		Floating Offshore Wind 2050 Overnight Costs ($/kW)				
		$5,178	$4,458	$3,468	$3,017	$2,296
Wave Energy 2050 Overnight Costs ($/kW)	$1,733	88.69	88.73	116.39	147.53	166.69
	$1,462	85.31	84.93	113.09	143.20	167.37
	$1,333	86.43	86.49	108.53	135.88	166.25
	$760	84.87	84.07	104.28	129.92	162.09
	$618	85.44	85.00	100.68	125.73	159.05

Collocation

Table 14: Number of sites chosen for collocation in 2050 in each scenario. Source data are provided as a Source Data file.

		Floating Offshore Wind 2050 Overnight Costs ($/kW)				
		$5,178	$4,458	$3,468	$3,017	$2,296
Wave Energy 2050 Overnight Costs ($/kW)	$1,733	0	0	1	2	2
	$1,462	0	2	5	3	2
	$1,333	0	2	7	8	6
	$760	1	5	13	10	14
	$618	2	10	26	28	26

Table 15: Percent of installed offshore wind and/or wave energy farms that have collocation of both technologies in 2050 in each scenario. Source data are provided as a Source Data file.

		Floating Offshore Wind 2050 Overnight Costs ($/kW)				
		$5,178	$4,458	$3,468	$3,017	$2,296
Wave Energy 2050 Overnight Costs ($/kW)	$1,733	0.00	0.00	2.94	4.88	4.00
	$1,462	0.00	6.90	12.20	6.98	3.92
	$1,333	0.00	4.65	13.73	14.29	10.91
	$760	1.59	6.94	15.85	12.50	18.92
	$618	2.06	9.52	21.49	23.33	23.64

Table 16: Sites which SWITCH chooses to collocate offshore wind and wave energy in 10 or more scenarios (2050). Source data are provided as a Source Data file.

Site Name	State	Latitude	Longitude	WECC Load Zone	Wind Turbine Type	# of Scenarios that Choose Site for Collocation
Capetown 5	CA	40.440	-125.363	CA_PGE_N	float	15
Capetown 3	CA	40.440	-124.448	CA_PGE_N	fixed	14
Capetown 2	CA	40.474	-124.480	CA_PGE_N	fixed	13
Brookings Call Area	OR	42.160	-124.816	OR_W	float	11
Sixes 2	OR	42.836	-124.946	OR_W	float	10
Capetown 4	CA	40.440	-125.051	CA_PGE_N	float	8
Capetown 1	CA	40.478	-124.432	CA_PGE_N	fixed	8
Wedderburn	OR	42.434	-124.532	OR_W	fixed	8
Sixes 1	OR	42.836	-124.654	OR_W	float	7
Lompoc 8	CA	34.076	-120.459	CA_PGE_S	fixed	6
Heceta Beach 3	OR	44.015	-124.371	OR_W	float	6
Denmark	OR	42.886	-124.620	OR_W	float	5
Heceta Beach 2	OR	44.131	-124.624	OR_W	float	5
Heceta Beach 1	OR	44.135	-124.168	OR_W	fixed	5

Table 17: WECC load zone abbreviation descriptions for load zones with the most frequently collocated sites. Source data are provided as a Source Data file.

WECC Load Zone Abbreviation	Description
CA_PGE_N	California Pacific Gas and Electric North
OR_W	Oregon/ Washington
CA_PGE_S	California Pacific Gas and Electric South

System Costs

Table 18: Total system itemized costs in each scenario, in net present value summed across all periods (Billion USD). Source data are provided as a Source Data file.

Scenario	Fuel Costs	Energy Storage Fixed Costs	Transmission Fixed Costs	Generation Variable O&M Costs	Generation Fixed Costs	Total System Cost
1	200.62	38.88	16.92	29.19	362.46	648.07
2	200.73	37.11	16.44	29.23	363.33	646.83
3	201.16	36.11	15.99	29.31	362.46	645.03
4	201.28	34.97	15.81	29.33	360.69	642.07
5	201.08	34.39	15.70	29.29	358.55	639.00
6	200.61	38.42	17.01	29.18	362.57	647.79
7	200.72	36.75	16.54	29.22	363.36	646.58
8	201.14	35.72	16.05	29.31	362.57	644.80
9	201.21	34.65	15.90	29.32	360.77	641.85
10	200.99	34.29	15.65	29.28	358.56	638.78
11	200.70	29.91	19.28	29.15	365.24	644.28
12	200.74	29.90	18.92	29.15	364.95	643.66
13	200.97	29.89	18.24	29.19	363.93	642.23
14	200.98	29.03	18.11	29.19	362.32	639.62
15	201.08	29.51	17.72	29.21	359.18	636.71
16	200.49	25.13	21.32	29.07	363.83	639.84
17	200.38	25.23	21.18	29.05	363.52	639.36
18	200.53	25.57	20.27	29.09	362.89	638.36
19	200.54	25.30	19.79	29.08	361.30	636.01
20	200.55	25.28	19.50	29.07	358.97	633.37
21	198.77	21.15	22.54	28.79	356.27	627.52
22	198.84	20.70	22.53	28.83	356.33	627.23
23	198.79	20.37	22.41	28.82	356.20	626.59
24	198.66	19.44	21.84	28.81	356.22	624.97
25	198.73	19.27	21.60	28.79	354.27	622.67

5 Supplementary Model Information: SWITCH Data

This section of the Supplementary Information contains information about the data used in the SWITCH model, including geographic and temporal data, generator data, transmission data, costs, load data, and policies.

To optimize long-term energy system buildout under the cost target scenarios of this study, we use the capacity expansion model SWITCH. SWITCH is an open-source model with high spatial and temporal resolution designed to plan a system with high levels of renewable resources [11], and has been used to evaluate system expansion in several case studies of the Western U.S. [12], [13], [14]. We build on the SWITCH 2.0 (Python) version from a recent study [11], and use an academic license of the Gurobi solver [15], to evaluate the optimal generation and transmission capacity expansion decisions for the Western Interconnect or Western Electricity Coordinating Council (WECC) region out to 2050. SWITCH makes investment and operations decisions for four time periods and a sample of hours per period between 2020 and 2050. The objective function minimizes the expected value of the total net present value of generation and transmission operations and investment. We provide a detailed description and full mathematical formulation of the SWITCH model in section 6.

Geographic and Temporal Resolution

The WECC study area of this analysis is divided up into 50 “load zone” regions (Figure 12), covering all of parts of Washington, Oregon, California, Arizona, Nevada, New Mexico, Utah, Idaho, Montana, Wyoming, Colorado, and Texas in the US; British Columbia and Alberta in Canada; and the northern portion of Baja California in Mexico. Load zone boundaries were used from prior SWITCH-WECC analyses [4, 5], which constructed the regions based on state lines, North American Electric Reliability Corporation (NERC) control areas, utility service territory boundaries, and high-population metropolitan areas. Some utility service territories were divided into multiple zones if there was a large amount of high-voltage transmission connectivity existing within the same service territory (i.e. CA PGE BAY, CA PGE CEN, CA PGE S, CA PGE N for PG&E). The boundaries were also constructed to represent zones that are unlikely to have transmission congestion within the zone (since only transmission between zones is included), and between which there has historically been transmission congestion (reflecting pathways where additional transmission may be needed).

The temporal resolution of the study includes investment periods covering four decadal durations: 2020, 2030, 2040, and 2050, but the analysis focuses on 2050 (covering 2046 – 2055). The duration of these investment periods reflects the planning horizons typically used in the long-term planning process of a utility, and the length of time often needed to plan and build generation and transmission infrastructure. As mentioned in the work presented, to represent each period as ten-year periods, we sample every month in 2020, 2030, 2040, and 2050, two days per month (median and peak load days) and every four hours per day (12 months x 2 days/month x 6 hour/day = 144 hours). Peak days have a weight of 1 and median days of (n – 1) where n is the number of days of that month, and this represents a full month.

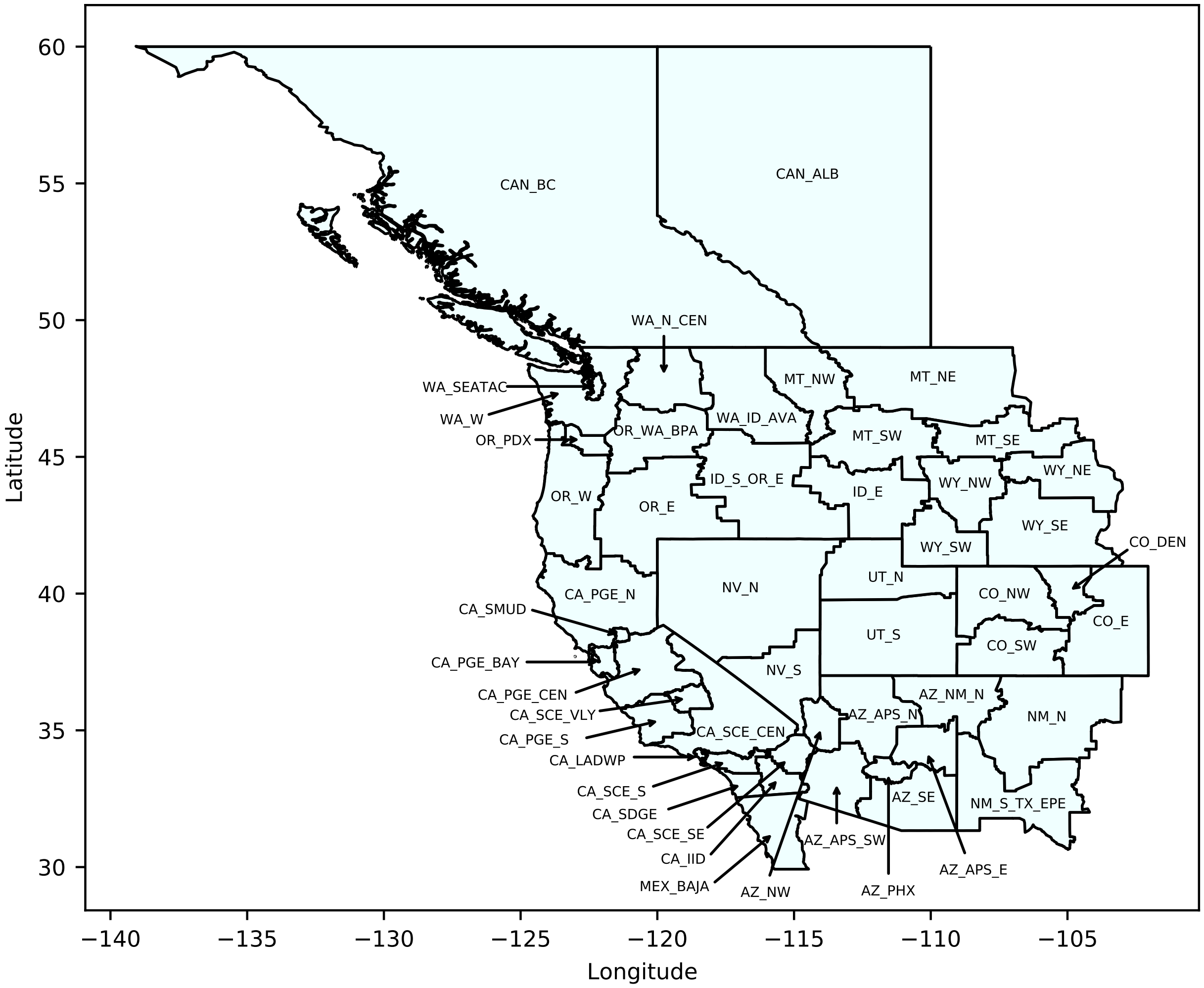

Figure 12: SWITCH-WECC load zone boundaries.

Generators

Existing Generation Data

As inputs into SWITCH, we include the list of individual generators that are existing and/or are planned for the WECC region. For the US portion of WECC, we extract the list of generators and their characteristics (such as location, fuel source, generating technology, online year, and retirement year if any) from the Energy Information Administration (EIA) Form 860 [16]. At the time of the data collection (in late 2019/early 2020), the latest complete year of data was the 2018 EIA Form 860. We include generators from this form that are flagged with any of these statuses: operating, standby/backup, new unit under construction, cold standby, out of service but will be returned to service, construction complete but not yet in commercial operation, not under construction but site prep underway, and under

construction. Generators are assigned to load zones based on their latitude and longitude.

The EIA Form 860 also includes planned retirement years for generators, if known. Generators are not allowed to operate beyond their expected lifetime, and therefore planned retirement years are used to calculate the lifetime of the plant to be included in the SWITCH runs. Monthly data was used from more recent Form 860 data to confirm that there were no additional planned retirements. For generators with no known retirement years, we assign a maximum lifetime based on the generating technology (Table 20).

For thermal generators, we join the list of generators from Form 860 with the monthly generation and fuel use from the EIA Form 923, for the available years 2004 – 2018 [17]. We use the historical monthly generation and fuel use from Form 923 to calculate the second-best (or second-lowest) heat rate (MMBtu/MWh) for each generator, avoiding any outliers from the best heat rate. The heat rate is used in the SWITCH dispatch optimization to calculate the fuel use and associated variable cost and emissions from dispatching thermal generators.

Hydropower generators are constrained to generate at their average historical monthly capacity factor [13]. We extract the monthly generation by generator from 2004 – 2018 (the most recent complete data at the time of the analysis) from the Form 923. For each generator, we calculate the average power for each month (monthly generation/hours in month) and estimate that the proxy for minimum flows for each generator is the power generated at half the average monthly level. For the SWITCH base case (without climate change), for each generator we calculate the average monthly power (and minimum flow power) over the years 2004 – 2018. These average values are repeated for all future investment periods of the SWITCH simulation (from 2020 – 2050). Monthly hydropower capacity factors by generator for the Canadian load zones (British Columbia and Alberta) are used from the prior SWITCH database, based on data from Statistics Canada Tables [13].

The existing generator set for the Canadian and Mexican load zones are used from the data previously compiled for prior SWITCH-WECC analysis, originally from the WECC Transmission Expansion Planning Policy Committee database of generators, and were not updated for this study [13], [14].

Candidate Generators

One of the key decision variables in SWITCH is the capacity investment of generation, out of a set of candidate generators with specific generating technologies and fuel sources, load zone locations, and other physical and financial generating characteristics. We use the dataset of candidate generators that was previously compiled in prior SWITCH-WECC analyses [12], [13], [14].

Candidate onshore and offshore wind generators were derived based on wind power output from a gridded 3TIER Western Wind and Solar Integration Study dataset [18] and a gridded Canadian wind developer dataset, and a selection of prime sites based on criteria including high wind energy density, and proximity to transmission [13]. A portion of candidate generators were screened out in California if they were in “Category 3, high environmental

risk" locations, which include areas legally excluded for development, protected areas with ecological or social value, conservation regions, and prime agricultural land [19].

Candidate solar generators include Residential PV (rooftop PV on homes), Commercial PV (rooftop PV on commercial buildings), Central PV (utility-scale), and Concentrating Solar Power with and without storage (solar thermal trough systems with or without thermal energy storage). Distributed Residential and Commercial PV candidate generation had been derived based on a gridded population density dataset, solar insolation data from NREL's (now deprecated) Solar Prospector tool, and assumptions on rooftop area and solar cell characteristics [13]. Available land and capacity for Central PV and Concentrating Solar Power candidate generators were screened based on land exclusion criteria (including national parks, wildlife areas, and steep terrain), solar insolation from the System Advisor Model from the National Renewable Energy Laboratory [20], and assumptions on the solar technology characteristics [13].

To simulate the dispatch of wind and solar generators, we use an exogenous dataset of hourly capacity factors by generator that had been constructed in prior SWITCH analyses [6, 8]. For wind generators, hourly capacity factors for the candidate generator set were calculated from the 3TIER Western Wind and Solar Integration Study wind speed dataset [18] using idealized turbine power curves. For solar generators, hourly capacity factors for the candidate generator set are calculated from the System Advisor, using data from 2006 (consistent with the base weather year underlying the load profiles) [20]. Central PV and onshore wind generators with capacity-weighted average capacity factors below the 75th percentile for their technology were screened out to only have the candidate set among a computationally tractable, and commercially viable, set of higher-quality resource sites [13]. For existing solar and wind generators, we average the hourly capacity factors for all solar and wind generators, respectively, in each load zone, and assign all the generators in that load zone the average capacity factor for the given technology.

Biogas (from landfill, wastewater treatment plants, and manure) candidate generator availability is derived from an assessment of the technical resource/feed stock availability [21]. Bioliquid generators are allowed to be reinstalled in their current locations but no new bioliquid plants are assumed. No new biomass (bio solid) candidate generators are assumed, but cogeneration bio solid generation is allowed to be reinstalled at the end of its lifetime [13]. Candidate geothermal generators are based on the current locations and capacity of existing plants that may be reinstalled after retirement [13]. We assume there is no candidate hydropower generation.

Natural gas combined cycle generators and combustion turbines do not have imposed maximum capacity limits. Coal generators are not allowed to be installed in California but are otherwise allowed to expand without capacity limits. Cogeneration plants (with gas combined cycle or combustion turbine plants) are given the option to be reinstalled at current locations after reaching their maximum age, at current capacity limits. We assume that there is no nuclear generation available for new, candidate generation.

Battery storage is available for installation in all load zones and investment periods, without capacity limits. An AC-DC-AC storage efficiency of 85%, a lifetime of 10 years, and

a variable O&M cost of 0 is assumed, consistent with prior SWITCH analyses [13]. Table 19 summarizes the maximum capacity available for the set of candidate generators, and the capacity installed for existing generators.

Table 19: WECC total available capacity of existing and candidate generation. Source data are provided as a Source Data file.

Existing or Candidate Generator	Energy Source	Generation Technology	Capacity Limit (GW)
Existing Generator	Biogas	Biogas	0.2
		Biogas Internal Combustion Engine	0.3
		Biogas Internal Combustion Engine Cogen	0.2
		Biogas Steam Turbine	0.1
	Bio Liquid	Bio Liquid Steam Turbine Cogen	0.4
	Bio Solid	Bio Solid Steam Turbine	0.6
		Bio Solid Steam Turbine Cogen	0.5
	Coal	Coal Steam Turbine	42.2
		Coal Steam Turbine Cogen	0.3
	Distillate Fuel Oil	Distillate Fuel Oil Combustion Turbine	0.4
		Distillate Fuel Oil Internal Combustion Engine	0.4
	Electricity	Battery Storage	1.1
	Gas	CCGT	54.8
		CCGT Cogen	7.7
		Gas Combustion Turbine	23.6
		Gas Combustion Turbine Cogen	4.3
		Gas Internal Combustion Engine	1.1
		Gas Internal Combustion Engine Cogen	0.4

Table 19: (continued)

Existing or Candidate Generator	Energy Source	Generation Technology	Capacity Limit (GW)
		Gas Steam Turbine	16.0
		Gas Steam Turbine Cogen	0.3
		Other Turbine	0.0
	Geothermal	Geothermal	4.5
	Residual Fuel Oil	Gas Combustion Turbine	0.2
	Solar	Central PV	22.0
		Concentrating Solar Power Trough, No Storage	0.4
		Steam Turbine	1.4
	Uranium	Nuclear	7.7
	Waste Heat	Other Turbine	0.0
		Steam Turbine	0.2
	Water	Hydropower	63.3
		Pumped Storage	4.3
	Wind	Onshore Wind	27.3
	**Total**		**286.0**
Candidate Generators	Biogas	Biogas	0.0
		Biogas Internal Combustion Engine Cogen	0.1
	Bio Liquid	Bio Liquid Steam Turbine Cogen	0.3
	Bio Solid	Bio Solid Steam Turbine Cogen	0.4
	Coal	Coal Integrated Gasification Combined Cycle (IGCC)	Not limited, except not allowed in CA
		Coal Steam Turbine	Not limited, except not allowed in CA
		Coal Steam Turbine Cogen	0.7
	Electricity	Battery Storage	Not limited

Table 19: (continued)

Existing or Candidate Generator	Energy Source	Generation Technology	Capacity Limit (GW)
	Gas	Combined Cycle Gas Turbine (CCGT)	Not limited
		CCGT Cogen	6.9
		Gas Combustion Turbine	Not limited
		Gas Combustion Turbine Cogen	4.6
		Gas Internal Combustion Engine Cogen	0.1
		Gas Steam Turbine Cogen	0.3
	Geothermal	Geothermal	11.1
	Solar	Central PV	3,270.2
		Commercial PV	52.7
		Concentrating Solar Power Trough, 6 hours storage	3,695.3
		Concentrating Solar Power Trough, No Storage	5,362.5
		Residential PV	125.3
	Wind	Offshore Wind	6.4
		Onshore Wind	521.4
	**Total**		**13,058.2**

Technology Assumptions On Lifetime, Capacity Factors, Efficiencies

For all existing generators and candidate generators, we assume the following default technology assumptions based on the generating technology and fuel source (Table 20), primarily based on the technology and cost assumptions from Black & Veatch [22] and a CEC Cost of Generation Report collected in the prior SWITCH analyses [6, 7] and converted to \$2018, with some technology values (natural gas combined cycle, natural gas turbine, battery, wind, solar PV, solar CSP, and geothermal) updated based on NREL's 2020 Annual Technology Baseline [23]. For existing generators with known planned retirement years, a specific lifetime is calculated.

Table 20: Technology assumptions for existing and candidate generators. Source data are provided as a Source Data file.

Fuel Source	Generating Technology	Lifetime (Years)	Forced Outage Rate (%)	Scheduled Outage Rate (%)
Biogas	Biogas	20	4%	6%
	Biogas Internal Combustion Engine	20	4%	6%
	Biogas Internal Combustion Engine Cogen	20	11%	4%
	Biogas Steam Turbine	20	13%	9%
Bio Liquid	Bio Liquid Steam Turbine Cogen	40	13%	9%
Bio Solid	Bio Solid Steam Turbine	20	4%	6%
	Bio Solid Steam Turbine Cogen	40	13%	9%
Coal	Coal IGCC	40	8%	12%
	Coal Steam Turbine	40	4%	6%
	Coal Steam Turbine Cogen	40	6%	10%
Distillate Fuel Oil	Distillate Fuel Oil Combustion Turbine	40	4%	6%
	Distillate Fuel Oil Internal Combustion Engine	20	4%	6%
Electricity	Battery Storage	10	2%	1%
Gas	CCGT	40	4%	6%
	CCGT Cogen	20	4%	6%
	Gas Combustion Turbine	40	4%	6%
	Gas Combustion Turbine Cogen	20	3%	5%
	Gas Internal Combustion Engine	20	4%	6%
	Gas Internal Combustion Engine Cogen	20	3%	5%

Table 20: (continued)

Fuel Source	Generating Technology	Lifetime (Years)	Forced Outage Rate (%)	Scheduled Outage Rate (%)
	Gas Steam Turbine	60	4%	6%
	Gas Steam Turbine Cogen	40	13%	9%
	Other Turbine	40	4%	6%
Geothermal	Geothermal	20	0%	0%
Residual Fuel Oil	Gas Combustion Turbine	40	4%	6%
Solar	Central PV	20	0%	0%
	Commercial PV	20	0%	0%
	Concentrating Solar Power Trough 6h Storage	20	6%	0%
	Concentrating Solar Power Trough No Storage	20	0%	0%
	Residential PV	20	0%	0%
	Steam Turbine	20	4%	6%
Uranium	Nuclear	80	4%	6%
Waste Heat	Other Turbine	40	4%	6%
	Steam Turbine	40	4%	6%
Water	Hydropower	200	5%	5%
	Pumped Storage	200	5%	5%
Wind	Offshore Wind	20	5%	1%
	Onshore Wind	20	0%	0%

Transmission

Existing and Candidate Transmission

The SWITCH optimization includes the construction of new transmission lines and the operations of existing and new transmission as decision variables. The model includes a set of 105 existing aggregated transmission lines between load areas within the WECC (Figure 13), based on a prior SWITCH analyses that aggregated the thermal limits of individual high-voltage lines between load areas from a Ventyx purchased dataset and Federal Energy Regulatory Commission (FERC) data [13]. New transmission capacity may be added to existing transmission corridors or constructed between 21 adjacent load zone pairs where there is currently no transmission. The line length assumed is 1.3 times the straight-line

distance between the largest substations in each load zone, based on a prior analysis that calculated this as the average ratio between line length and straight-line distance between transmission substations in WECC [13]. For every 100 miles of distance, a 1% efficiency loss is assumed, based on typical losses for high-voltage transmission [24]. For both existing and newly constructed transmission lines, the maximum power transfer on each line is the thermal limit multiplied by a derating factor. The derating factor is from a prior SWITCH analysis and is meant to capture the combined effect of stability concerns, loop flows, voltage concerns, power factors less than unity, and overloading of individual transmission lines within the bundle, that are difficult to model in detail in the linear model. The factors are 0.59 for alternating current (AC) lines, and 0.91 for direct current (DC) lines, of which there are only two in our analysis [13].

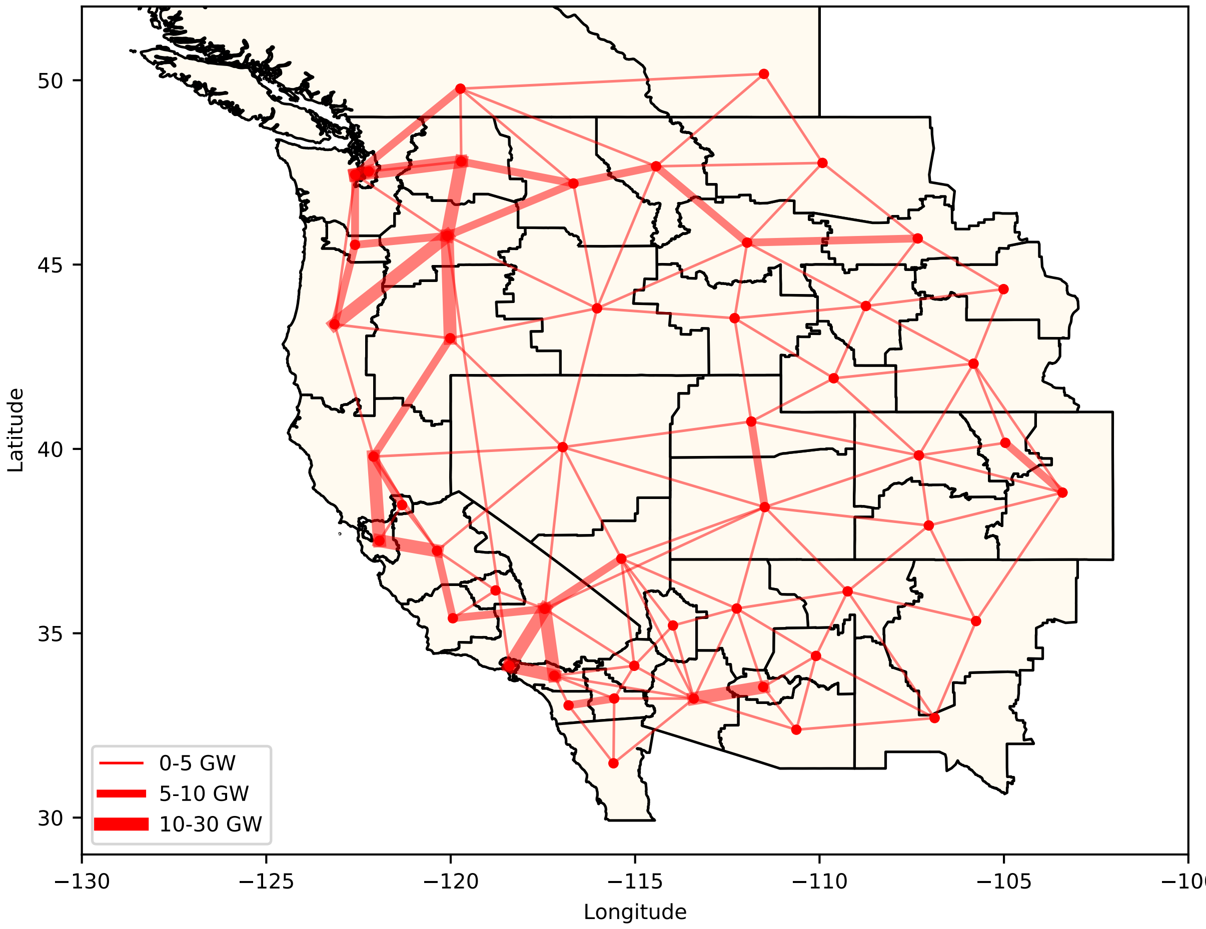

Figure 13: Transmission topology and transmission rating category considered in 50 load zones for the WECC region used in the SWITCH-WECC model. Transmission lines are categorized as follows: 5 GW (0-5], 10 GW (5-10], and 30 GW (10-30]. Source data are provided as a Source Data file.

Costs

Overnight Capital Cost, Variable O&M Cost, Fixed O&M Cost

The SWITCH optimization includes several types of costs in the decision to invest and/or operate in generation. Costs for candidate generators include overnight capital cost (applied to the built capacity), fixed O&M costs per MW-year of operations, and variable O&M costs per MWh of operation. Mature technologies (biogas, bioliquid, biosolid, coal, gas cogeneration, gas steam turbine) are assumed to have their real costs stay constant over time, whereas other technologies are assumed to decrease costs over time with technology improvements and economies of scale. Capital, fixed O&M, and variable O&M costs by generator technology type originate primarily from Black & Veatch estimates for the mature technologies [6, 13]. For technologies with changing costs over time (battery, solar PV, solar CSP, wind, geothermal, gas CCGT, gas CT), we compile cost data from NREL's 2020 Annual Technology Baseline database using the "Moderate Scenario" which is based on the of median projections from the literature [23]. For wind and central solar PV, we also account for lower overnight costs in the first investment period from the Production Tax Credit and Investment Tax Credit, respectively [25, 19]. For battery storage, we separate out the capital costs into $/MW (balance of system battery cost) and $/MWh (battery pack cost which would be multiplied by the storage duration hours) costs [23]. The costs we use assume a four-hour duration battery. The average costs by decadal investment period, energy source, and technology are in Table 21. Capital costs for existing generators are considered sunk costs and we do not include them in the total system costs; they do not affect future investment decisions. Variable O&M costs for existing generators are set to be the same as for candidate generators.

Table 21: Average capital, fixed O&M, and variable O&M costs by investment period for candidate generators ($2018). Source data are provided as a Source Data file.

Energy Source	Generation Technology	Investment Period	Overnight capital cost ($/MW)	Fixed O&M cost ($/MW-yr)	Variable O&M cost ($/MWh)
Bio Gas	Bio Gas	2020	2,118,354	64,380	9.20
		2030	2,118,354	64,380	9.20
		2040	2,118,354	64,380	9.20
		2050	2,118,354	64,380	9.20
	Bio Gas Internal Combustion Engine Cogen	2020	1,588,766	48,285	16.00
		2030	1,588,766	48,285	16.00
		2040	1,588,766	48,285	16.00
		2050	1,588,766	48,285	16.00

Table 21: (continued)

Energy Source	Generation Technology	Investment Period	Overnight capital cost ($/MW)	Fixed O&M cost ($/MW-year)	Variable O&M cost ($/MWh)
Bio Liquid	Bio Liquid Steam Turbine Cogen	2020	3,225,737	80,012	16.80
		2030	3,225,737	80,012	16.80
		2040	3,225,737	80,012	16.80
		2050	3,225,737	80,012	16.80
Bio Solid	Bio Solid Steam Turbine Cogen	2020	3,225,737	80,012	16.80
		2030	3,225,737	80,012	16.80
		2040	3,225,737	80,012	16.80
		2050	3,225,737	80,012	16.80
Coal	Coal Integrated Gasification Combined Cycle (IGCC)	2020	4,503,135	34,924	7.34
		2030	4,503,135	34,924	7.34
		2040	4,503,135	34,924	7.34
		2050	4,503,135	34,924	7.34
	Coal Steam Turbine	2020	3,245,403	25,828	4.57
		2030	3,245,403	25,828	4.57
		2040	3,245,403	25,828	4.57
		2050	3,245,403	25,828	4.57
	Coal Steam Turbine Cogen	2020	2,434,058	19,371	4.20
		2030	2,434,058	19,371	4.20
		2040	2,434,058	19,371	4.20
		2050	2,434,058	19,371	4.20
Electricity	Battery Storage	2020	414,708	32,043	0.0
		2030	150,026	20,982	0.0
		2040	126,912	17,749	0.0
		2050	113,216	15,834	0.0
Gas	CCGT	2020	1,115,074	11,708	4.50
		2030	981,532	12,863	4.50
		2040	947,742	12,863	4.50
		2050	924,780	12,863	4.50
	CCGT Cogen	2020	1,035,945	5,314	4.12
		2030	1,035,945	5,314	4.12
		2040	1,035,945	5,314	4.12
		2050	1,035,945	5,314	4.12
	Gas Combustion Turbine	2020	914,407	10,297	2.16
		2030	896,544	11,395	2.16
		2040	864,990	11,395	2.16
		2050	844,878	11,395	2.16

Table 21: (continued)

Energy Source	Generation Technology	Investment Period	Overnight capital cost ($/MW)	Fixed O&M cost ($/MW-year)	Variable O&M cost ($/MWh)
	Gas Combustion Turbine Cogen	2020	548,292	4,430	33.58
		2030	548,292	4,430	33.58
		2040	548,292	4,430	33.58
		2050	548,292	4,430	33.58
	Gas Internal Combustion Engine Cogen	2020	565,136	4,430	33.58
		2030	565,136	4,430	33.58
		2040	565,136	4,430	33.58
		2050	565,136	4,430	33.58
	Gas Steam Turbine Cogen	2020	418,841	26,717	4.46
		2030	418,841	26,717	4.46
		2040	418,841	26,717	4.46
		2050	418,841	26,717	4.46
Geothermal	Geothermal	2020	7,753,680	143,173	0.0
		2030	7,658,660	173,766	0.0
		2040	7,237,757	173,105	0.0
		2050	6,970,164	173,105	0.0
Solar	Central PV	2020	1,088,245	22,665	0.0
		2030	876,956	10,270	0.0
		2040	758,373	8,882	0.0
		2050	702,651	8,229	0.0
	Commercial PV	2020	1,550,322	20,415	0.0
		2030	1,089,723	7,813	0.0
		2040	910,568	6,529	0.0
		2050	819,995	5,879	0.0
	CSP Trough 6h Storage	2020	6,845,931	63,634	3.59
		2030	4,915,309	54,132	3.59
		2040	4,205,950	52,447	3.59
		2050	4,023,815	52,447	3.59
	CSP Trough No Storage	2020	5,074,725	56,149	0.0
		2030	4,661,582	56,149	0.0
		2040	4,248,663	56,149	0.0
		2050	3,937,151	56,149	0.0
	Residential PV	2020	2,078,361	25,068	0.0
		2030	1,256,559	9,424	0.0
		2040	984,462	7,384	0.0
		2050	884,353	6,633	0.0

Table 21: (continued)

Energy Source	Generation Technology	Investment Period	Overnight capital cost ($/MW)	Fixed O&M cost ($/MW-year)	Variable O&M cost ($/MWh)
Wind	Offshore Wind	2020	3,959,165	112,298	0.0
		2030	2,744,489	112,298	0.0
		2040	2,401,870	112,298	0.0
		2050	2,226,776	112,298	0.0
	Onshore Wind	2020	1,393,460	47,047	0.0
		2030	1,247,480	38,867	0.0
		2040	1,120,978	35,883	0.0
		2050	1,042,433	33,692	0.0

Connection Costs

In addition to capital costs for the construction of a generator itself, for candidate generators we also add a connection cost to reflect the expense of connecting to the grid. This connection cost originally derived from EIA data and compiled in a prior SWITCH analysis [13] is either a "generic" cost if the generator is not located at a specific site (for gas, coal, biosolid, biogas, or battery storage), or is calculated for a specific site (for onshore wind, offshore wind, geothermal, central PV, and CSP with and without storage). The "generic" cost includes the cost of building a substation ($80,000/MW in $2018) and a small transmission line to the substation ($31,000 in $2018). Site specific connection costs include a transmission line cost of $1,200 per MW per km based on the distance from the site to the substation, and the cost of the substation. The cost for underwater cable for offshore wind is $6000 per MW per km. There is no connection cost applied for existing generation.

Fuel Costs and Emissions

Fuel costs are applied to non-renewable generators (natural gas, coal, fuel oil, uranium) and originate from the EIA's Annual Energy Outlook (AEO) compiled from a prior WECC analysis [14]. Gas costs differ by load zone based differences in regional market prices and the wellhead price [13]. Table 22 shows the average costs for each decadal investment period, across all the load zones, in $2018. The fuel costs for bio solid generators are based on supply curves derived based on estimates of the economically feasible volumes of biomass feedstock available by load zone and different fuel price tiers [13].

Table 22: Average fuel costs across load zones, by investment period ($/MMBtu in $2018). Source data are provided as a Source Data file.

Fuel Source	2020	2030	2040	2050
Bio Liquid	0.01	0.01	0.01	0.01
Coal	2.13	2.17	2.27	2.33
Distillate Fuel Oil	18.55	22.89	25.34	27.64
Gas	4.32	5.17	5.39	6.34
Residual Fuel Oil	11.88	16.34	18.30	20.44
Uranium	0.68	0.88	1.15	1.53

Transmission Costs

The cost of building new transmission lines is calculated as the product of a line length, a base $/MW/km cost, a terrain multiplier that reflects the topography differences that make a line more expensive to construct, and an economic multiplier that represents differences in labor, permitting, and other "soft" costs between WECC load zones. We assume a base $960/MW-km cost, which is the cost for constructing a 500 kV line in the WECC from the ReEDS capacity expansion model database ($1,347/MW-mile in $2010 dollars, converted to $/MW-km and $2018 dollars) [24]. For the economic multiplier, for all lines within California we use a factor of 2.25, between California and other WECC load zones we use a factor of 1.125, and within other WECC load zones we use a factor of 1 (keeping the base cost as it is) from the ReEDS documentation [24]. Steeper terrain and urban land area increase transmission construction costs. Terrain multipliers come from a prior SWITCH GIS analysis that overlaid the transmission line paths over a gridded dataset of terrain-dependent transmission costs, that had been derived from the slope and the land cover in the WECC region [13]. The multipliers range from 0.7 to 3.4. Transmission lines also have a fixed O&M cost applied to reflect upkeep costs for lines, which is assumed to be 3% of capital costs from the prior SWITCH analysis.

Load

The future load assumed in this analysis was developed in a prior study and represents a case of high energy efficiency and building electrification, as well as increased adoption of Zero Emissions Vehicles (ZEVs), primarily from electric vehicles [14]. The load forecast achieves a doubling of the rate of energy efficiency by 2030 in California, compliant with the state's SB 350 legislative targets, aggressive building electrification starting in 2020, growing industry electrification, and approximately 125,000 GWh in electricity demand from transportation. Hourly demand profiles from 2006 (consistent with the weather-year used for calculating solar and wind capacity factors) from FERC Form 714 and a dataset procured from ITRON were used as a base from which demand projects (residential, commercial, industrial, transportation) were created and scaled by sector to meet states' policy targets and reflect population growth [26]. Where detailed state/province-level load forecasts with

state efficiency, electrification, and population estimates were available (including California, Washington, Oregon, British Columbia, and Alberta) load zone forecasts were scaled to those projections; otherwise forecasts were scaled to the EIA's Annual Energy Outlook projections in the prior SWITCH analysis [7, 18]. In the 2017 Annual Energy Outlook Electric projections used, population growth across the U.S. is on average 0.6% annually, based on the U.S. Census Bureau's mid-case projections at the time [27]. Electric vehicles are assumed to charge in an "unmanaged" way (without smart charging or time-of-use rates), based on charging profiles developed with an agent-based mobility model BEAM [19, 20]. Planning Reserves We assume that the load zones in the WECC region must meet a planning reserve margin of 15%, that is, the model is required to build capacity to meet 115% of the peak load in each reserve area. The reserve requirement is applied by reserve area; utilities like Southern California Edison (SCE) and Pacific Gas & Electric (PG&E) that span multiple load zones have a combined reserve requirement across their total region. All generator technologies are assumed to be eligible to provide capacity towards meeting the planning reserve requirement. Thermal generators contribute their nameplate capacity, solar and wind generators contribute as much as their capacity factor during the peak hour, hydropower generators contribute as much as their monthly capacity factors for all hours of that month, battery storage contributes as much as it discharges during the peak hour, and net transmission imports contribute as much as the flows during the peak hour.

Policies

Renewable Portfolio Standards

We include state Renewable Portfolio Standard (RPS) policies in SWITCH as a constraint requiring a fraction of electricity demand be generated by renewable generators. Qualifying RPS-eligible technologies include solar PV and CSP, wind, geothermal, biogas, bio solid, bio liquid, and hydropower. We include the annual schedule of RPS requirements by state [28]. Because of computational tractability, the RPS constraint is calculated as a WECC-wide load-weighted average requirement (total RPS-eligible generation annually must equal or exceed the sum-product of annual load for each load zone and the RPS percentage requirement for that load zone), rather than accounting for the stocks, flows, and transmission imports of renewable power from specific generators.

Carbon Cap

A constraint on carbon emissions from generators can be imposed in SWITCH by load zone and investment period. In this analysis, the carbon cap that is enforced is zero-emissions by 2050 in the entire WECC.

To measure compliance with this constraint, SWITCH tracks the emissions from each generator and aggregates to the load zone, based on emissions intensity for each fuel source [29] (Table 24).

Table 23: WECC-wide and California carbon cap average by investment period. Source data are provided as a Source Data file.

Investment Period	Carbon cap (tons CO_2 per year)	Carbon cap in CA only (tons CO_2 per year)
2050	0	0

A constraint on carbon emissions from generators can be imposed in SWITCH by load zone and investment period. In this analysis, the carbon cap that is enforced is zero-emissions by 2050 in the entire WECC.

To measure compliance with this constraint, SWITCH tracks the emissions from each generator and aggregates to the load zone, based on emissions intensity for each fuel source [29] (Table 24).

Table 24: Emissions intensity of fuel-based energy sources (tons CO2/BTU). Source data are provided as a Source Data file.

Fuel	CO2 Intensity of Fuel (tons CO2/BTU)
Biogas	0
Bio Liquid	0
Bio Solid	0
Coal	0.09552
Distillate Fuel Oil	0.07315
Gas	0.05306
Residual Fuel Oil	0.0788
Uranium	0

6 Supplementary SWITCH Mathematical Formulation

This section of the Supplementary Information contains the objective function, variables, parameters, and constraints for the modules that were implemented for the SWITCH Western Electricity Coordinating Council (WECC) model we use in this paper. For a full description of the SWITCH model refer to [30]. This document was adapted and synthesized from the Supplementary Information in [30], courtesy of J. Johnston, R. Henriquez-Auba, B. Maluenda and M. Fripp.

Objective Function

The objective function minimizes the total cost of investments and operations (as net present value):

$$\min \sum_{p \in \mathcal{P}} d_p \left\{ \sum_{c^{\mathrm{f}} \in \mathcal{C}^{\mathrm{fixed}}} c_p^{\mathrm{f}} + \sum_{t \in \mathcal{T}_p} w_t^{\mathrm{year}} \sum_{c^{\mathrm{v}} \in \mathcal{C}^{\mathrm{var}}} c_t^{\mathrm{v}} \right\} \tag{1}$$

Where $\mathcal{P}$ is the set of periods in the optimization, $\mathcal{C}^{\mathrm{fixed}}$ is the set of fixed costs, $\mathcal{C}^{\mathrm{var}}$ is the set of variable costs, $\mathcal{T}_p$ is the set of timepoints in the optimization, and p, c^{f}, t and c^{v} are respective elements in those sets. The term c_p^{f} is the fixed cost that occurs during period p, c_t^{v} is the variable cost per timepoint t, w_t^{year} scales costs from a sampled timepoint to an annualized value, and d_p is the discount factor that converts the costs to net present value.

Operational Constraints

Power balance

Power injection and withdrawal must be equal in each load zone for all timepoints.

$$\sum_{p^{\mathrm{i}} \in \mathcal{P}^{\mathrm{inject}}} p_{z,t}^{\mathrm{i}} = \sum_{p^{\mathrm{w}} \in \mathcal{P}^{\mathrm{withdraw}}} p_{z,t}^{\mathrm{w}}, \qquad \forall z \in \mathcal{Z}, \forall t \in \mathcal{T} \tag{2}$$

Depending on the SWITCH modules used, power injection $\mathcal{P}^{\mathrm{inject}}$ includes power output $P_{g,t}$ for every generation project g located in load zone z and incoming transmission flows to the load zone z, $F_{\ell_z^{\mathrm{in}},t}$. Power withdrawal $\mathcal{P}^{\mathrm{withdraw}}$ typically includes electricity loads $l_{z,t}$ and outgoing transmission flows $F_{\ell_z^{\mathrm{out}},t}$.

Dispatch

The power generation constraint per generator g is the following:

$$0 \leq P_{g,t} \leq \eta_{g,t} K_{g,p}^{\mathrm{G}}, \qquad \forall \ell \in \mathcal{L}, p \in \mathcal{P}, \forall t \in \mathcal{T}_p \tag{3}$$

For firm generators, $\eta_{g,t}$ is constant and represents average outage rates. For intermittent generators, $\eta_{g,t}$ also represents the renewable source's capacity factor at a timepoint t.

The constraint on transmission limits flows $F_{\ell,t}$ through a line ℓ based on capacity $K_{\ell,p}^{\mathrm{L}}$ and derated by the factor η_ℓ^{L}:

$$0 \leq F_{\ell,t} \leq \eta_\ell^{\mathrm{L}} K_{\ell,p}^{\mathrm{L}}, \qquad \forall \ell \in \mathcal{L}, p \in \mathcal{P}, \forall t \in \mathcal{T}_p \tag{4}$$

Investment Constraints

Generation projects that have a cap, $\overline{k^{\mathrm{G}}}_g$, on their maximum capacity installed are constrained by 5. Eqs. (6) and (8) represent cumulative installed capacity for generation projects $K^{\mathrm{G}}_{g,p}$ and transmission lines $K^{\mathrm{L}}_{\ell,p}$ until period p. As such, they are defined as the sum of previous capacity additions $B^{\mathrm{G}}_{g,p'}$ and $B^{\mathrm{L}}_{\ell,p'}$, including existing infrastructure. The set $\mathcal{P}^{\mathrm{on}}_{g,p}$ corresponds to the set of all periods when capacity of type g could be built and still be in service in period p. Eqs. (7) and (9) fix the investment decisions of some generation projects and transmission lines over some periods $\mathcal{P}^{\mathrm{G}}_g$ and $\mathcal{P}^{\mathrm{L}}_\ell$ with predetermined values specified in input files (which is the case of existing, or pre-planned capacity).

$$0 \leq K^{\mathrm{G}}_{g,p} \leq \overline{k^{\mathrm{G}}}_g, \qquad \forall g \in \mathcal{G}^{\mathrm{rc}}, p \in \mathcal{P} \tag{5}$$

$$K^{\mathrm{G}}_{g,p} = \sum_{p' \in \mathcal{P}^{\mathrm{on}}_{g,p}} B^{\mathrm{G}}_{g,p'}, \qquad \forall g \in \mathcal{G}, \forall p \in \mathcal{P} \cup \{p_0\} \tag{6}$$

$$B^{\mathrm{G}}_{g,p} = b^{\mathrm{G}}_{g,p}, \qquad \forall g \in \mathcal{G}, \forall p \in \mathcal{P}^{\mathrm{G}}_g \tag{7}$$

$$K^{\mathrm{L}}_{\ell,p} = \sum_{p' \in \mathcal{P} \cup \{p_0\} : p' \leq p} B^{\mathrm{L}}_{p',k}, \qquad \forall \ell \in \mathcal{L}, \forall p \in \mathcal{P} \tag{8}$$

$$B^{\mathrm{L}}_{\ell,p} = b^{\mathrm{L}}_{\ell,p}, \qquad \forall \ell \in \mathcal{L}, \forall p \in \mathcal{P}^{\mathrm{L}}_\ell \tag{9}$$

SWITCH Modules

Treatment of time

SWITCH uses three levels of time scales: timepoints, timeseries and period. Timepoints represent the highest time granularity in the optimization. Depending on the user of SWITCH, a timepoint can represent one hour or a set of hours. Timeseries are a set of consecutive timepoints. Examples of timeseries would be a day, a few days, a week, a month, or a year. A period is a set of consecutive years where investments can be made. Table 25 summarizes the time components in SWITCH. The code for this module can be found in `switch_model.timescales`.

Table 25: Model components defined in the `timescales` module.

Type	Symbol	Component Name	Description
Set	$\mathcal{P}$	PERIODS	Set of all investment periods, indexed by p.
Parameter	st_p	period_start[p]	Year in which period p begins.
Parameter	y_p	period_length_years[p]	Length in years of period p.
Set	$\mathcal{S}$	TIMESERIES	Set of all time series. Indexed by s.
Subset	$\mathcal{S}_p$	TS_IN_PERIOD[p]	Subset of time series that fall in period p.
Parameter	num_s	ts_num_tps[s]	Number of time points in time series s.
Parameter	Δ_s^{S}	ts_duration_of_tp[s]	Duration in hours of each time point in time series s. Used for short-term thermodynamics such as energy storage calculations.
Parameter	Δ_t^{T}	tp_duration_hrs[t]	Duration in hours of time point t (equal to Δ_s^{S} for the corresponding timeseries).
Parameter	w_t^{period}	tp_weight[t]	Weight of timepoint t within simulation (hours).
Parameter	w_t^{year}	tp_weight_in_year[t]	Weight of timepoint t within its year (hours/year).
Parameter	θ_s	ts_scale_to_period[s]	Number of times a time series s (or equivalent conditions) occurs in its period. Used statistically for sample weighting for economics, pollution and long-term energy demand.
Set	$\mathcal{T}$	TIMEPOINTS	Set of all time points, indexed by t.
Subset	$\mathcal{T}_s$	TPS_IN_TS[s]	Subset of time points that fall in time series s.
Subset	$\mathcal{T}_p$	TPS_IN_PERIOD[p]	Subset of time points that fall in period p.

The weight of a timepoint's within a period is given by

$$w_t^{\mathrm{period}} = \Delta_t^{\mathrm{T}} \cdot \theta_s, \qquad \forall p \in \mathcal{P}, \forall s \in \mathcal{S}_p, \forall t \in \mathcal{T}_s.$$

This reflects the fact that each timepoint represents Δ_t^{T} hours within its timeseries, and each timeseries is treated as recurring θ_s times in its period.

Financial components

The code for this module can be found in `switch_model.financials`.

Table 26: Model components defined in the `financials` module.

Type	Symbol	Component Name	Description
Parameter	r	discount_rate	Annual real discount rate used to convert future dollars to present.
Parameter	i	interest_rate	Annual real interest rate used to finance investments.
Parameter	baseyear	base_financial_year	Base financial year in which future costs will be converted to net present value via discount rate r.
Set	$\mathcal{C}^{\mathrm{fixed}}$	Cost_Components_Per_Period	Fixed cost components that contribute to the total cost in the cost-minimizing objective function.
Set	$\mathcal{C}^{\mathrm{var}}$	Cost_Components_Per_TP	Variable cost components that contribute to the total cost in the cost-minimizing objective function.

Load zones and power injection/withdrawal

The code for this module can be found in `switch_model.balancing.load_zones`.

Table 27: Model components defined in the `balancing.load_zones` module.

Type	Symbol	Component Name	Description
Set	$\mathcal{Z}$	LOAD_ZONES	Set of all load zones, indexed by z.
Parameter	$l_{z,t}$	zone_demand_mw[z, t]	Demand in MW at zone z at time point t.
Subset	$\mathcal{Z}^{\text{peak}}$	EXTERNAL_ COINCIDENT_ PEAK_DEMAND_ ZONE_PERIODS	Subset of load zones, period pairs for which and expected peak load has bbeen provided.
Parameter	$l^{\text{peak}}_{z,p}$	zone_expected_ coincident_peak_ demand[z,p]	Expected peak load demand in zone z in period p, $(z,p) \in \mathcal{Z}^{\text{peak}}$ (optional).
Set	$\mathcal{P}^{\text{inject}}$	Zone_Power_ Injections	Model components that inject power to the central bus of each load zone.
Set	$\mathcal{P}^{\text{withdraw}}$	Zone_Power_ Withdrawals	Model components that withdraw power from the central bus of each load zone.

Energy sources

The code for this module can be found in `switch_model.energy_sources.properties`.

Table 28: Model components defined in the `energy_sources.properties` module.

Type	Symbol	Component Name	Description
Set	$\mathcal{E}$	ENERGY_SOURCES	Set of all energy sources, indexed by f.
Subset	$\mathcal{E}^{\text{F}}$	FUELS	Subset of all fuel-based energy sources.
Subset	$\mathcal{E}^{\text{R}}$	NON_FUEL_ENERGY_ SOURCES	Subset of all non-fuel energy sources.
Parameter	$\xi_f, f \in \mathcal{E}^{\text{F}}$	f_co2_intensity[f]	Direct emissions of CO2 of a fuel in tCO2/MMBtu.
Parameter	$\mu_f, f \in \mathcal{E}$	f_upstream_co2_intensity[f]	Emissions attributable to an energy-source before it is consumed in tCO2/MMBtu.

Investment components
The code for this module can be found in `switch_model.generators.core.build`.

Table 29: Model components defined in the `generators.core.build` module.

Type	Symbol	Component Name	Description
Set	$\mathcal{G}$	GENERATION_PROJECTS	Set of all generation projects, indexed by g.
Subset	$\mathcal{G}^{\mathrm{B}}$	BASELOAD_GENS	Subset of all generation projects that are baseload.
Subset	$\mathcal{G}^{\mathrm{F}}$	FUEL_BASED_GENS	Subset of all generation projects that use fuels.
Subset	$\mathcal{G}^{\mathrm{R}}$	VARIABLE_GENS	Subset of all generation projects that are variable renewable.
Subset	$\mathcal{G}^{\mathrm{rc}}$	CAPACITY_LIMITED_GENS	Subset of all generation projects that are resource constrained.
Subset	$\mathcal{G}^{\mathrm{Z}}_{z}$	GENS_IN_ZONE[z]	Subset of all generation projects that are located in load zone z.
Subset	$\mathcal{G}^{\mathrm{T}}_{\mathrm{tech}}$	GENS_BY_TECHNOLOGY[tech]	Subset of all generation projects that are of the generation technology, tech.
Parameter	b^{unit}_{g}	gen_unit_size[g]	Size of individual generating units within project g (optional).
Subset	$\mathcal{G}^{\mathrm{unit}}$	DISCRETELY_SIZED_GENS	Subset of generation projects for which a discrete unit size $\mathrm{b}^{\mathrm{unit}}_{g}$ has been specified.
Subset	$\mathcal{E}^{\mathrm{F}}_{g}$	FUELS_FOR_GEN[g]	Subset of fuels that can be used by generator g.
Parameter	$\mathrm{ly}^{\mathrm{G}}_{g}$	gen_max_age[g]	Operational lifetime of capacity added to generation project g (years). Capital costs are also amortized over this period.
Parameter	$b^{\mathrm{G}}_{g,p}$	gen_predetermined_cap[g,p]	Predetermined addition of capacity in project g during period p (MW).
Set	$\mathcal{GP}^{\mathrm{B}}$	GEN_BLD_YRS	Set of tuples of generation projects g and periods p in which capacity may be added.
Subset	$\mathcal{GP}^{\mathrm{D}}$	PREDETERMINED_GEN_BLD_YRS	Subset of generation projects g and periods p for which the capacity additions are predetermined (may include years before the main study).
Subset	$\mathcal{P}^{\mathrm{on}}_{g,p}$	BLD_YRS_FOR_GEN_PERIOD[g,p]	Set of periods (including p) when capacity of type g could have been built and still be in service in period p. This excludes capacity that would be retired before period p.
Subset	$\mathcal{P}^{\mathrm{on}}_{g}$	PERIODS_FOR_GEN[g]	Subset of periods when generation project g may have capacity online. This excludes periods before g can be built or after it must be retired.
Parameter	$\overline{k^{\mathrm{G}}}_{g}$	gen_capacity_limit_mw[g]	Maximum allowed capacity for project g (MW).
Variable	$B^{\mathrm{G}}_{g,p}$	BuildGen[g,p]	Amount of capacity built (added) in project g in period p; $(g,p) \in \mathcal{GP}^{\mathrm{B}}$.
Variable	$K^{\mathrm{G}}_{g,p}$	GenCapacity[g,p]	Cumulative capacity of project g as of period p.
Parameter	$\hat{c}^{\mathrm{G,inv}}_{g}$	gen_overnight_cost[g, p]	Overnight capital cost per MW to add capacity to project g in period p; $(g,p) \in \mathcal{GP}^{\mathrm{B}}$.
Parameter	$\hat{c}^{\mathrm{G,upg}}_{g}$	gen_connect_cost_per_mw[g]	Overnight cost of grid upgrades to support the project g, per MW installed.
Parameter	$c^{\mathrm{G,fix}}_{g}$	gen_fixed_om[g,p]	Fixed operation and maintenance costs per MW of capacity per year, for capacity added to project g in period p; $(g,p) \in \mathcal{GP}^{\mathrm{B}}$. This cost recurs every year until the capacity retires ($\mathrm{ly}^{\mathrm{G}}_{g}$ years).
Parameter	$c^{\mathrm{G,var}}_{g}$	gen_variable_om[g]	Variable operation and maintenance costs per MWh of power produced by project g.
Parameter	h_g	gen_full_load_heat_rate[g]	Full load heat rate (inverse of thermal efficiency), in MMBtu per MWh. May be supplemented by part-load heat rates in generators.core.commit.fuel_use.

Dispatch components

The code for this module can be found in `switch_model.generators.build.dispatch` and `switch_model.generators.core.no_commit`.

Table 30: Model components defined in the `generators.core.dispatch` module.

Type	Symbol	Name	Description
Subset	$\mathcal{T}_g^{\text{on}}$	TPS_FOR_GEN[g]	Subset of timepoints when generation project g may have capacity online. Corresponds to $\mathcal{P}_g^{\text{on}}$ defined in generators.core.build.
Subset	$\mathcal{GT}^{\text{on}}$	GEN_TPS	Set of tuples of generator g and timepoint t when capacity can be online. $\mathcal{GT}^{\text{on}} = \{(g,t) : g \in \mathcal{G} \text{ and } t \in \mathcal{T}_g^{\text{on}}\}$.
Subset	$\mathcal{T}_{g,p}^{\text{on}}$	TPS_FOR_GEN_IN_PERIOD[g,p]	Subset of timepoints when generation project $g \in \mathcal{G}$ has capacity available during period $p \in \mathcal{P}$. Includes all timepoints in p if $(g,p) \in \mathcal{GT}^{\text{on}}$, otherwise the empty set.
Variable	$P_{g,t}$	DispatchGen[g,t]	Average power in MW produced by project g during timepoint t.
Variable	$R_{g,t,f}$	GenFuelUseRate[g,t,f]	Rate of use of fuel $f \in \mathcal{E}_g^{\text{F}}$ by project $g \in \mathcal{G}^{\text{F}}$ during timepoint t (in MMBtu/h). Each generator may use multiple fuels.
Parameter	η_g	gen_forced_outage_rate[g]	Fraction of time a project $g \in \mathcal{G}$ is expected to be available (used to de-rate for forced outages).
Parameter	$\eta_{g,t}$	gen_max_capacity_factor[g,t]	Maximum possible output from renewable project $g \in \mathcal{G}^{\text{R}}$ in timepoint t (per-unit).

Table 31: Model components defined in the `generators.core.no_commit` module.

Type	Symbol	Component Name	Description
Variable	$P_{g,p}^{\text{B}}$	DispatchBaseloadByPeriod[g,p]	Amount of power to produce from baseload generator $g \in \mathcal{G}^{\text{B}}$ during all timepoints in period $p \in \mathcal{P}_g^{\text{on}}$ (MW)

The constraints are the following:

$$P_{g,t} = P_{g,p(t)}^{\text{B}}, \qquad \forall g \in \mathcal{G}^{\text{B}}, \forall t \in \mathcal{T}_g^{\text{on}} \tag{10}$$

$$0 \leq P_{g,t} \leq \eta_g K_{g,p(t)}^{\text{G}}, \qquad \forall g \in \mathcal{G} - \mathcal{G}^{\text{R}}, \forall t \in \mathcal{T}_g^{\text{on}} \tag{11}$$

$$0 \leq P_{g,t} \leq \eta_g \eta_{g,t} K_{g,p(t)}^{\text{G}}, \qquad \forall g \in \mathcal{G}^{\text{R}}, \forall t \in \mathcal{T}_g^{\text{on}} \tag{12}$$

$$\sum_{f \in \mathcal{E}_g^{\text{F}}} R_{g,t,f} = h_g P_{g,t}, \qquad \forall g \in \mathcal{G}^{\text{F}}, \forall t \in \mathcal{T}_g^{\text{on}} \tag{13}$$

Fuel costs

The code for this module can be found in `switch_model.energy_sources.fuel_costs.markets`.
Fuels costs can be modeled as a yearly cost per fuel for each load zone, and as a supply curve for a regional market (set of load zones).

Table 32: Model components defined in the `energy_sources.fuel_costs.simple` module.

Type	Symbol	Component Name	Description
Parameter	$c_{z,f,p}^{\text{fuel}}$	fuel_cost[z,f,p]	Cost per MMBtu for fuel f in load zone z during period p.
Set	$\mathcal{F}^{\text{unav}}$	GEN_TP_FUELS_UNAVAILABLE	Set of tuples of (project g, timepoint t, fuel f) where fuel f is not available (i.e., user has not specified a cost).

Total fuel costs for each zone and timepoint are calculated as

$$\sum_{g \in \mathcal{G}_z^{\text{Z}} : t \in \mathcal{T}_g^{\text{on}}} \sum_{f \in \mathcal{E}_g^{\text{F}}} c_{z,f,p(t)}^{\text{fuel}} \times R_{g,t,f}, \qquad \forall z \in \mathcal{Z}, t \in \mathcal{T}. \tag{14}$$

Table 33: Model components defined in the `energy_sources.fuel_costs.markets` module.

Type	Symbol	Component Name	Description
Set	$\mathcal{M}$	REGIONAL_FUEL_MARKETS	Set of all regional fuel markets (rfm), indexed by m.
Parameter	f^{M}_m	rfm_fuel[m]	Type of fuel that is sold in the regional fuel market m.
Subset	$\mathcal{Z}^{\mathrm{M}}_m$	ZONES_IN_RFM[m]	Set of all load zones served by the regional fuel market m.
Set	$\Sigma_{m,p}$	SUPPLY_TIERS_FOR_RFM_PERIOD	Set of supply tiers (i.e., complete supply curve) for a given regional fuel market m and period p, indexed by σ.
Set	$\mathcal{MP}\Sigma$	RFM_SUPPLY_TIERS	Set of valid tuples of regional fuel market m, period p and supply curve tier σ; $\mathcal{MP}\Sigma = \{(m,p,\sigma) : m \in \mathcal{M}, p \in \mathcal{P}, \sigma \in \Sigma_{m,p}\}$
Set	$\mathcal{F}^{\mathrm{unav}}$	GEN_TP_FUELS_UNAVAILABLE	Set of tuples of (project g, timepoint t, fuel f) where fuel f is not available.
Parameter	$c^{\mathrm{fuel}}_{m,p,\sigma}$	rfm_supply_tier_cost[m,p,σ]	Cost of a fuel in a particular tier of a supply curve $(m,p,\sigma) \in \mathcal{MP}\Sigma$.
Parameter	$\mathrm{limit}_{m,p,\sigma}$	rfm_supply_tier_limit[m,p,σ]	Annual limit of fuel available for a particular tier in the supply curve $(m,p,\sigma) \in \mathcal{MP}\Sigma$.
Variable	$R^{\mathrm{tier}}_{m,p,\sigma}$	ConsumeFuelTier[m,p,σ,]	The annual rate of fuel consumption in each tier of a supply curve, $(m,p,\sigma) \in \mathcal{MP}\Sigma$, in MMBtu/year.

Constraints regarding the tier limits and linkage with power production are defined in this module:

$$0 \le R^{\mathrm{tier}}_{m,p,\sigma} \le \mathrm{limit}_{m,p,\sigma}, \qquad \forall (m,p,\sigma) \in \mathcal{MP}\Sigma \tag{15}$$

$$\sum_{\sigma \in \Sigma_{m,p}} R^{\mathrm{tier}}_{m,p,\sigma} = \sum_{z \in \mathcal{Z}^{\mathrm{M}}_m} \sum_{g \in \mathcal{G}^{\mathrm{Z}}_z : f^{\mathrm{M}}_m \in \mathcal{E}^{\mathrm{F}}_g} \sum_{t \in \mathcal{T}_p \cap \mathcal{T}^{\mathrm{on}}_g} w^{\mathrm{year}}_t R_{g,t,f}, \qquad \forall m \in \mathcal{M}, \forall p \in \mathcal{P} \tag{16}$$

$$R_{g,t,f} = 0, \qquad \forall (g,t,f) \in \mathcal{F}^{\mathrm{unav}} \tag{17}$$

Total fuel costs of all tiers of a supply curve from all regional fuel markets during period p are calculated as:

$$\mathrm{AnnualFuelCosts}_p = \sum_{m \in \mathcal{M}} \sum_{\sigma \in \Sigma_{m,p}} c^{\mathrm{fuel}}_{m,p,\sigma} \cdot R^{\mathrm{tier}}_{m,p,\sigma}, \qquad \forall p \in \mathcal{P}$$

These are added to the set $\mathcal{C}^{\mathrm{fixed}}$ in order to be considered in the objective function (1).

Transmission components

The code for this section can be found in `switch_model.transmission.transport.build` and `switch_model.transmission.transport.dispatch`.

Table 34: Model components defined in the `transmission.transport.build` module.

Type	Symbol	Component Name	Description
Set	$\mathcal{L}$	TRANSMISSION_LINES	Set of all transmission corridors, indexed by ℓ.
Parameter	ζ_ℓ^1	trans_lz1[l]	Load zone at the start of corridor ℓ.
Parameter	ζ_ℓ^2	trans_lz2[l]	Load zone at the end of corridor ℓ.
Parameter	km_ℓ	trans_length_km[l]	Length in km of transmission corridor $\ell \in \mathcal{L}$.
Parameter	η_ℓ^L	trans_derating_factor[l]	Overall derating factor for transmission corridor ℓ that can reflect forced outage rates, stability or contingency limitations.
Parameter	$\eta_\ell^{\mathrm{L,ef}}$	trans_efficiency[l]	Efficiency; proportion of power sent through corridor ℓ that reaches the other end.
Subset	$\mathcal{LB}$	TRANS_BLD_YRS	Set of transmission corridors ℓ and periods p where capacity can be added
Parameter	b_ℓ^{L}	existing_trans_cap[l]	Transfer capability existing in corridor ℓ prior to the start of the study.
Variable	$B_{\ell,p}^{\mathrm{L}}$	BuildTx[l,p]	Transfer capability added in corridor ℓ during period p (MW); $(\ell, p) \in \mathcal{LB}$.
Expression	$K_{\ell,p}^{\mathrm{L}}$	TxCapacityNameplate[l, p]	Cumulative transfer capability through transmission corridor ℓ as of period p.
Parameter	$\hat{c}^{\mathrm{L}}$	trans_capital_cost_per_mw_km	Generic cost of expanding transfer capability in base year dollars per MW per km.
Parameter	α_ℓ	trans_terrain_multiplier	Cost multiplier for expanding capacity on a specific corridor ℓ.
Parameter	β	trans_fixed_om_fraction	Describes the fixed O&M costs per year as a fraction of capital costs.
Parameter	ly^{L}	trans_lifetime_yrs	Lifetime over which capital costs are amortized (years). Note that capacity is assumed to continue in service after this date, with the same annual payment (equivalent to automatically reconstructing capacity when it retires).
Set	$\mathcal{L}^{\mathrm{D}}$	DIRECTIONAL_TX	Set of directed transmission corridors. It consists of the tuples $(\zeta_\ell^1, \zeta_\ell^2)$ and $(\zeta_\ell^2, \zeta_\ell^1)$, for all $\ell \in \mathcal{L}$. Elements of this set refer to flows from the first zone of the tuple to the second zone.
Set	$\mathcal{L}_z^{\mathrm{D}}$	TX_CONNECTIONS_TO_ZONE[z]	Set of directed transmission corridors that flow into zone z (i.e., the second element is equal to z).

The annualized cost (capital and O&M) per MW of capacity in transmission corridor ℓ is:

$$c_\ell^{\mathrm{L,inv}} = \left(\frac{i}{1-(1-i)^{-\mathrm{ly}^{\mathrm{L}}}}\right)\hat{c}^{\mathrm{L}} \cdot \alpha_\ell \cdot \mathrm{km}_\ell$$
$$c_\ell^{\mathrm{L,fix}} = \beta c_\ell^{\mathrm{L,inv}}$$

These costs are multiplied by the installed transmission capacity as of each future period, $K_{\ell,p}^{\mathrm{L}}$, then discounted to the base year, and added to the set $\mathcal{C}^{\mathrm{fixed}}$ for inclusion in the objective function (1).

The installed capability until period p is calculated as:

$$K_{\ell,p}^{\mathrm{L}} = b_\ell^{\mathrm{L}} + \sum_{p':(\ell,p')\in\mathcal{LB} \text{ and } p'\le p} B_{\ell,p'}^{\mathrm{L}}, \qquad \forall \ell \in \mathcal{L}, \forall p \in \mathcal{P}. \tag{18}$$

Table 35: Model components defined in the `transmission.transport.dispatch` module.

Type	Symbol	Component Name	Description
Variable	$F_{z,z',t}$	DispatchTx[z,z_,t]	Power flow through a directed corridor $(z, z') \in \mathcal{L}^{\mathrm{D}}$ (i.e., from zone z to zone z') during timepoint t.
Expression	$F^{\mathrm{net}}_{z,t}$	TXPowerNet[z,t]	Net power inflow to zone z from all other zones during timepoint t

The constraint of maximum flow through lines is defined as:

$$0 \leq F_{z,z',t} \leq \eta^{\mathrm{L}}_{\ell(z,z')} K_{\ell(z,z'),p}, \qquad \forall (z,z') \in \mathcal{L}^{\mathrm{D}}, \forall p \in \mathcal{P}, \forall t \in \mathcal{T}_p, \tag{19}$$

where $\ell(z, z')$ identifies the transmission corridor $\ell \in \mathcal{L}$ corresponding to the directed corridor $(z, z') \in \mathcal{L}^{\mathrm{D}}$. With this approach, net inflows to zone z from all other zones can be calculated as total inflows minus total outflows:

$$F^{\mathrm{net}}_{z,t} = \sum_{z':(z',z) \in \mathcal{L}^{\mathrm{D}}} \eta^{\mathrm{L,ef}}_{\ell(z',z)} F_{z',z,t} - \sum_{z':(z,z') \in \mathcal{L}^{\mathrm{D}}} F_{z,z',t} \qquad \forall z \in \mathcal{Z}. \tag{20}$$

$F^{\mathrm{net}}_{z,t}$ is added to the set of power-injecting components $\mathcal{P}^{\mathrm{inject}}$ for inclusion in the power balance equation (2).

Hydropower components

The code for this module can be found in `switch_model.generators.extensions.hydro_simple`.

Table 36: Model components defined in the `generators.extensions.hydro_simple` module.

Type	Symbol	Component Name	Description
Subset	$\mathcal{G}^{\mathrm{H}}$	HYDRO_GENS	Subset of all hydro-based generation projects.
Parameter	$p^{\mathrm{h,min}}_{g,s}$	hydro_min_flow_mw[g,s]	Minimum flow level, expressed as electrical MW, for all timepoints of timeseries s.
Parameter	$p^{\mathrm{h,avg}}_{g,s}$	hydro_avg_flow_mw[g,s]	Average flow level, in electrical MW, that must be achieved during timeseries s.

Power dispatch must exceed the minimum level for all timepoints, and the average power production during a time series must be equal to the average flow rate:

$$P_{g,t} \geq p^{\mathrm{h,min}}_{g,s}, \qquad \forall g \in \mathcal{G}^{\mathrm{H}}, \forall s \in \mathcal{S}, \forall t \in \mathcal{T}_s. \tag{21}$$

$$\frac{1}{\mathrm{num}_s} \cdot \sum_{t \in \mathcal{T}_s} P_{g,t} = p^{\mathrm{h,avg}}_{g,s}, \qquad \forall g \in \mathcal{G}^{\mathrm{H}}, \forall s \in \mathcal{S}. \tag{22}$$

Storage components

The code for this module can be found in `switch_model.generators.extensions.storage`.

Table 37: Model components defined in the `generators.extensions.storage` module.

Type	Symbol	Component Name	Description
Subset	$\mathcal{G}^{\mathrm{S}}$	STORAGE_GENS	Subset of all generation projects that can store electricity for later discharge.
Subset	$\mathcal{GP}^{\mathrm{S}}$	STORAGE_GEN_BLD_YRS	Subset of all tuples of generation project g and period p when storage projects can be built; $\mathcal{GP}^{\mathrm{S}} = \{(g,p) : (g,p) \in \mathcal{GP}^{\mathrm{G}}$ and $g \in \mathcal{G}^{\mathrm{S}}\}$.
Parameter	η_g^{S}	gen_storage_efficiency[g]	Fraction of energy that is stored in the battery out of the total energy fetched from the grid.
Parameter	$\hat{c}_{g,p}^{\mathrm{S,inv}}$	gen_storage_energy_overnight_cost[g]	Overnight capital cost per MWh adding energy storage capacity (not power output) to storage project g in period p.
Parameter	$r_g^{\max}$	gen_store_to_release_ratio[g]	The maximum charging rate for storage project g, expressed as a ratio relative to the maximum power output rate.
Parameter	$r_g^{\mathrm{S,ep}}$	gen_storage_energy_to_power_ratio[g]	Fixed ratio of storage capacity to power rating (hours) for storage project g. Optional; if not specified, Switch optimizes the amount of energy storage capacity.
Parameter	$n_g^{\mathrm{S,max}}$	gen_storage_max_cycles_per_year[g]	Maximum amount of discharging allowed per year, for storage project g, expressed as a multiple of the installed storage capacity (optional).
Variable	$B_{g,p}^{\mathrm{S}}$	BuildStorageEnergy[g,p]	Amount of energy storage capacity to add to project g in period p, in MWh; $(g,p) \in \mathcal{GP}^{\mathrm{S}}$.
Variable	$K_{g,p}^{\mathrm{S}}$	StorageEnergyCapacity[g,p]	Cumulative capacity in MWh of storage project g at period p.
Variable	$C_{g,t}^{\mathrm{S}}$	ChargeStorage[g,t]	Decision of how much to charge a storage project g at time point t.
Variable	$P_{g,t}$	DispatchGen[g,t]	Decision of how much to discharge a storage project g at time point t, i.e., how much power to deliver to the grid (defined in `generators.core.build`).
Variable	$\overline{P_{g,t}}$	DispatchUpperLimit[g,t]	Maximum possible power production by project g at timepoint t, (defined by equation (11)).
Variable	$Z_{g,t}^{\mathrm{S}}$	StateOfCharge[g,t]	State of charge in MWh of storage project g at timepoint t.
Parameter	$\eta_g^{S,loss}$	gen_self_discharge_rate[g]	Fraction of the charge that is lost per day.
Parameter	$\eta_g^{S,out}$	gen_discharge_efficiency[g]	Discharging efficiency: the fraction of energy that reaches the grid out of the energy drawn from the battery.
Parameter	ϵ_g^S	gen_land_use_rate[g]	Ratio of land area usage to storage capacity (in $\frac{m^2}{MWh}$).
Expression	$L_{g,p}^S$	LandUseRate[g,p]	Land use of a project during a period in m^2.

Overnight costs $\hat{c}_{g,p}^{\mathrm{S,inv}}$ are annualized and added to the set $\mathcal{C}^{\mathrm{fixed}}$ to be part of the objective function (1).

Power used for charging/discharging is added to the set of withdrawals $\mathcal{P}^{\mathrm{withdraw}}$/ injections $\mathcal{P}^{\mathrm{inject}}$ in the balance equation (2).

The constraints introduced to model investment and operation of storage consider cumu-

lative energy storage capacity, charging limits, storage required, and cycle limits per year.

$$K^{\mathrm{S}}_{g,p} = \sum_{p' \in \mathcal{P}:\ p' \leq p} B^{\mathrm{S}}_{g,p'}, \qquad \forall g \in \mathcal{G}^{\mathrm{S}}, p \in \mathcal{P} \tag{23}$$

$$0 \leq C^{\mathrm{S}}_{g,t} \leq r^{\max}_{g} \overline{P_{g,t}}, \qquad \forall g \in \mathcal{G}^{\mathrm{S}}, t \in \mathcal{T}^{\mathrm{on}}_{g} \tag{24}$$

$$0 \leq Z^{\mathrm{S}}_{g,t} \leq K^{\mathrm{S}}_{g,p}, \qquad \forall g \in \mathcal{G}^{\mathrm{S}}, t \in \mathcal{T}^{\mathrm{on}}_{g} \tag{25}$$

$$\mathit{coeff} = \begin{cases} \Delta^{T}_{t} & \eta^{S,loss}_{g} = 0 \\ 24 \dfrac{(1-\eta^{S,loss}_{g})^{\frac{\Delta^{T}_{t}}{24}} - 1}{\ln(1-\eta^{S,loss}_{g})} & \eta^{S,loss}_{g} \neq 0 \end{cases} \qquad \forall g \in \mathcal{G}^{S}, t \in \mathcal{T}^{on}_{g} \tag{26}$$

$$\mathit{flow}_{g,t} = \eta^{S,in}_{g} C^{S}_{g,t} - \frac{1}{\eta^{S,out}_{g}} P_{g,t} \qquad \forall g \in \mathcal{G}^{S}, t \in \mathcal{T}^{on}_{g} \tag{27}$$

$$Z^{S}_{g,t} = (1-\eta^{S,loss}_{g})^{\frac{\Delta^{T}_{t}}{24}} Z^{S}_{g,t-1} + (\mathit{coeff})(\mathit{flow}_{g,t}) \qquad \forall g \in \mathcal{G}^{S}, t \in \mathcal{T}^{on}_{g} \tag{28}$$

$$B^{\mathrm{S}}_{g,p} = r^{\mathrm{S,ep}}_{g} B^{\mathrm{G}}_{g,p}, \qquad \forall (g,p) \in \mathcal{GP}^{\mathrm{S}} : r^{\mathrm{S,ep}}_{g} \text{ specified} \tag{29}$$

$$\sum_{t \in \mathcal{T}_p} P_{g,t} \Delta^{\mathrm{T}}_{t} \leq n^{\mathrm{S,max}}_{g} K^{\mathrm{S}}_{g,p} y_p, \qquad \forall (g,p) \in \mathcal{GP}^{\mathrm{S}} \tag{30}$$

Equations 26 and 28 calculate how the state of charge changes every timestep while considering losses. These equations are derived by solving the differential equation:

$$\frac{dZ}{dt} = -rZ + flow$$

where Z is the amount of energy in storage, r is the instantaneous rate of energy loss per unit time (non-zero), and $flow$ is the net rate of energy entering the storage. The full derivation is shown below. Note that in the limiting case that the rate of energy loss, $\eta^{S,loss}_{g}$, is zero, this equation becomes much simpler.

Equation 27 specifies the net rate of energy entering the storage as the difference in charging and discharging rates while considering efficiency ratios.

Equation 31 specifies the expression for the land use.

$$L^{S}_{g,p} = \epsilon^{S}_{g} K^{S}_{g,p} \tag{31}$$

Deriving equations 26 and 28

We derive equation 26 and 28 by solving the following differential equation.

$$\frac{dZ}{dt} = -rZ + flow$$

This equation represents the instantaneous change in the amount stored, $\frac{dZ}{dt}$, as a combination of the net flow into the storage ($flow$), and the energy lost in storage over time ($-rZ$). Note that r is the instantaneous decay rate which we will later replace with, η, the hourly decay rate provided in the parameters.

We solve the differential equation as follows.

If our decay rate is zero ($r = \eta = 0$), we find:

$$\frac{dZ}{dt} = \mathit{flow}$$
$$Z = Z_0 + \Delta t(\mathit{flow})$$

Otherwise,

$$\frac{dZ}{dt} = -rZ + flow$$
$$\frac{dZ}{-rZ + flow} = dt$$
$$\int_{Z_0}^{Z} \frac{dZ}{-rZ + flow} = \int_{t_0}^{t} dt$$
$$[\frac{ln(-rZ + flow)}{-r}]_{Z_0}^{Z} = \Delta t$$
$$ln(\frac{-rZ + flow}{-rZ_0 + flow}) = -r\Delta t$$
$$-rZ + flow = (-rZ_0 + flow)e^{-r\Delta t}$$
$$Z = (Z_0 - \frac{flow}{r})e^{-r\Delta t} + \frac{flow}{r}$$
$$Z = Z_0 e^{-r\Delta t} + \frac{1 - e^{-r\Delta t}}{r} flow$$

We now wish to replace the instantaneous decay rate r with the provided daily self discharge decay rate, η. We know that without flow, the amount stored after one day should be less (by η percent) than the amount at the start of the day (Z_0). This gives the following equation:

$$Z_0 e^{-r \times 24 \text{ hour}} = Z_0(1 - \eta)$$

Simplifying we find:

$$r = -\frac{1}{24} ln(1 - \eta)$$

We now substitute r with $-\frac{1}{24}ln(1-\eta)$ in our solution.

$$Z = Z_0 e^{-r\Delta t} + \frac{1-e^{-r\Delta t}}{r} flow$$
$$Z = Z_0 e^{\frac{1}{24}ln(1-\eta)\Delta t} + \frac{1-e^{\frac{1}{24}ln(1-\eta)\Delta t}}{-\frac{1}{24}\ln(1-\eta)} flow$$
$$Z = Z_0(1-\eta)^{\frac{\Delta t}{24}} + 24\frac{(1-\eta)^{\frac{\Delta t}{24}}-1}{\ln(1-\eta)} flow$$

Rearranging the above equations gives equation (26 and 28):

$$Z = (1-\eta)^{\frac{\Delta t}{24}} Z_0 + (\mathit{coeff})(\mathit{flow})$$
$$\mathit{coeff} = \begin{cases} \Delta t & \eta = 0 \\ 24\frac{(1-\eta)^{\frac{\Delta t}{24}}-1}{\ln(1-\eta)} & \eta \neq 0 \end{cases}$$

Carbon cap components

The code for this module can be found in `switch_model.policies.carbon_policies`.

Table 38: Model components defined in the `policies.carbon_policies` module.

Type	Symbol	Component Name	Description
Parameter	cap_p	carbon_cap_tco2_per_yr[p]	Carbon cap in tons of CO_2 per year during period p (defaults to ∞).
Parameter	c_p^{carb}	carbon_cost_dollar_per_tco2[p]	Carbon cost per ton of CO_2 in period p (defaults to \$0).

$$\text{AnnualEmissions}_p = \sum_{g\in\mathcal{G}^{\text{F}}} \sum_{f\in\mathcal{E}_g^{\text{F}}} \sum_{t\in\mathcal{T}_g^{on}\cap\mathcal{T}_p} \Delta_t^{\text{T}} R_{g,t,f} \times (\xi_f + \mu_f), \qquad \forall p \in \mathcal{P} \tag{32}$$

The constraint that enforces the carbon cap is:

$$\text{AnnualEmissions}_p \leq \text{cap}_p, \qquad \forall p \in \mathcal{P} \tag{33}$$

Minimum technology requirements

The code for this module can be found in `switch_model.policies.min_per_tech`.

Table 39: Model components defined in the `policies.min_per_tech` module.

Type	Symbol	Component Name	Description
Parameter	$\text{min}_{\text{tech},p}$	minimum_capacity_mw[tech,p]	Minimum amount of capacity (in MW) needed throughout the model for a given technology and period (defaults to 0).
Parameter	$\text{min}^{\text{energy}}_{\text{tech},p}$	minimum_energy_capacity_mwh[tech,p]	Minimum amount of energy capacity (in MWh) needed throughout the model for a given storage technology and period (defaults to $0).

$$\sum_{g \in \mathcal{G}^{\mathcal{T}}_{\text{tech}}} \mathcal{K}^{\mathcal{G}}_{g,p} \geq \text{min}_{\text{tech},p}, \qquad \forall p \in \mathcal{P}, \text{tech} \in \text{TECHS} \tag{34}$$

$$\sum_{g \in \mathcal{G}^{\mathcal{T}}_{\text{tech}} \cap \mathcal{G}^{\mathcal{S}}} \mathcal{K}^{\mathcal{S}}_{g,p} \geq \text{min}^{\text{energy}}_{\text{tech},p}, \qquad \forall p \in \mathcal{P}, \text{tech} \in \text{TECHS} \tag{35}$$

Enforcing a solar to wind capacity ratio

The code for this module can be found in `switch_model.policies.wind_to_solar_ratio`.

Table 40: Model components defined in the `policies.wind_to_solar_ratio` module.

Type	Symbol	Component Name	Description
Parameter	ratio_p	wind_to_solar_ratio[p]	Ratio of total wind capacity to solar capacity (defaults to 0 which is coded to mean the constraint is inactive).

In our implementation we enforce the following constraint in only one direction (i.e. greater than or less than) such that the constraint is forcing (this improves numerical performance). However, for all effective purposes our constraint is equivalent to:

$$\sum_{g \in \mathcal{G}^{\mathcal{T}}_{\text{tech1}}} \mathcal{K}^{\mathcal{G}}_{g,p} = \text{ratio}_p * \sum_{g \in \mathcal{G}^{\mathcal{T}}_{\text{tech2}}} \mathcal{K}^{\mathcal{G}}_{g,p}, \qquad \text{where tech1=Wind, tech2=Solar} \tag{36}$$

Bibliography

[1] Bureau of Ocean Energy Management (BOEM). *State Activities*. URL: https://www.boem.gov/renewable-energy/state-activities.

[2] National Marine Protected Areas Center. *The MPA Inventory*. 2020. URL: https://marineprotectedareas.noaa.gov/dataanalysis/mpainventory/.

[3] Office for Coastal Management. *Danger Zones and Restricted Areas*. 2023. URL: https://www.fisheries.noaa.gov/inport/item/48876.

[4] Dominique Evans-Bye. *States_shapefile*. July 21, 2015. URL: https://hub.arcgis.com/datasets/1b02c87f62d24508970dc1a6df80c98e_0/.

[5] New York State Energy Research {and} Development Authority (NYSERDA). *Offshore Wind Submarine Cabling Overview*. 21-14. 2021. URL: https://www.nyftwg.com/wp-content/uploads/2021/05/Offshore-Wind-Submarine-Cable-Report.pdf.

[6] National Renewable Energy Laboratory (NREL). *Loads Analysis of a Floating Offshore Wind Turbine Using Fully Coupled Simulation (Preprint)*. NREL/CP-500-41714. 2007. URL: https://digital.library.unt.edu/ark:/67531/metadc891165/m2/1/high_res_d/909454.pdf.

[7] Frank Pendleton. *100m Depth Contours*. 2017. URL: https://databasin.org/datasets/60f4698c750a48b5ba2bcd6808fd9388/.

[8] National Oceanic {and} Atmospheric Administration. *U.S. Maritime Limits & Boundaries*. 2013. URL: https://nauticalcharts.noaa.gov/data/us-maritime-limits-and-boundaries.html.

[9] National Renewable Energy Laboratory. *DOE's Water Power Technology Office's (WPTO) US Wave Dataset, GitHub*. URL: https://github.com/NREL/hsds-examples/blob/master/datasets/US_Wave.md.

[10] National Renewable Energy Laboratory. *DOE's Water Power Technology Office's (WPTO) US Wave Dataset, Registry of Open Data on AWS*. URL: https://registry.opendata.aws/wpto-pds-us-wave.

[11] J Johnston et al. "Switch 2.0: A modern platform for planning high-renewable power systems." In: *SoftwareX* 10 (2019), p. 100251.

[12] J. Nelson. “High-resolution modeling of the western North American power system demonstrates low-cost and low-carbon futures”. In: *Energy Policy* 43 (2012), pp. 436–447.

[13] A. Mileva et al. “Power system balancing for deep decarbonization of the electricity sector”. In: *Applied Energy* 162 (2016), pp. 1001–1009. ISSN: 0306-2619. DOI: `https://doi.org/10.1016/j.apenergy.2015.10.180`. URL: `http://www.sciencedirect.com/science/article/pii/S0306261915014300`.

[14] M. Wei. *Building a Healthier and More Robust Future: 2050 Low-Carbon Energy Scenarios for California*. 2017. URL: `https://www.energy.ca.gov/2019publications/CEC-500-2019-033/CEC-500-2019-033.pdf`.

[15] Gurobi. *Gurobi - The fastest solver*. URL: `https://www.gurobi.com/`.

[16] U.S. Energy Information Administration. *Form EIA-860 detailed data with previous form data (EIA-860A/860B)*. URL: `https://www.eia.gov/electricity/data/eia860/`.

[17] U.S. Energy Information Administration. *Form EIA-923 detailed data with previous form data (EIA-906/920*. URL: `https://www.eia.gov/electricity/data/eia923/`.

[18] National Renewable Energy Laboratory. *Western Wind Dataset*. URL: `https://www.nrel.gov/grid/western-wind-data.html`.

[19] G. C. Wu, M. S. Torn, and J. H. Williams. “Incorporating Land-Use Requirements and Environmental Constraints in Low-Carbon Electricity Planning for California”. In: *Environmental Science & Technology* 49.4 (2015). PMID: 25541644, pp. 2013–2021. DOI: `10.1021/es502979v`. URL: `https://doi.org/10.1021/es502979v`.

[20] “System Advisor Model (SAM)”. In: *National Renewable Energy Laboratory* (). URL: `https://sam.nrel.gov/`.

[21] A. Milbrandt. *A Geographic Perspective on the Current Biomass Resource Availability in the United States*. 2005. URL: `https://www.nrel.gov/docs/fy06osti/39181.pdf`.

[22] Black and Veatch. *Cost and Performance Data for Power Generation Technologies*. 2012. URL: `https://refman.energytransitionmodel.com/publications/1921`.

[23] National Renewable Energy Laboratory. “2020 Annual Technology Baseline”. In: *Annual Technology Baseline* (). URL: `https://atb.nrel.gov/electricity/2020/data.php`.

[24] W. Cole. *Regional Energy Deployment System (ReEDS) Model Documentation: Version 2019*. 2020. DOI: `10.2172/1606151.`. URL: `https://www.osti.gov/biblio/1606151`.

[25] DOE Office of Energy Efficiency {and} Renewable Energy. *WINDExchange: Production Tax Credit and Investment Tax Credit for Wind*. URL: `https://windexchange.energy.gov/projects/tax-credits`.

[26] Federal Energy Regulatory Commission. *Form No. 714 - Annual Electric Balancing Authority Area and Planning Area Report*. URL: https://www.ferc.gov/industries-data/electric/general-information/electric-industry-forms/form-no-714-annual-electric/data.

[27] U.S. Energy Information Administration. *Assumptions to the Annual Energy Outlook*. 2017. URL: https://www.eia.gov/outlooks/aeo/assumptions/pdf/0554(2017).pdf.

[28] National Conference of State Legislatures. *State Renewable Portfolio Standards and Goals*. Aug. 13, 2021. URL: https://www.ncsl.org/research/energy/renewable-portfolio-standards.aspx.

[29] U.S. Energy Information Administration. *Carbon Dioxide Emissions Coefficients*. URL: https://www.eia.gov/environment/emissions/co2_vol_mass.php.

[30] J. Johnston et al. “Switch 2.0: A modern platform for planning high-renewable power systems”. In: *SoftwareX* 10 (2019), p. 100251. ISSN: 2352-7110. DOI: https://doi.org/10.1016/j.softx.2019.100251. URL: http://www.sciencedirect.com/science/article/pii/S2352711018301547.
